# Panomics reveals patient individuality as the major driver of colorectal cancer progression

**DOI:** 10.1186/s12967-022-03855-0

**Published:** 2023-01-23

**Authors:** Friederike Praus, Axel Künstner, Thorben Sauer, Michael Kohl, Katharina Kern, Steffen Deichmann, Ákos Végvári, Tobias Keck, Hauke Busch, Jens K. Habermann, Timo Gemoll

**Affiliations:** 1 Section for Translational Surgical Oncology and Biobanking, Department of Surgery, University of Lübeck and University Hospital Schleswig-Holstein, Campus Lübeck, Ratzeburger Allee 160, 23538 Lübeck, Germany; 2 Medical Systems Biology Group, Lübeck Institute Für Experimental Dermatology, University of Lübeck, Campus Lübeck, 23538 Lübeck, Germany; 3 Department of Surgery, University Hospital Schleswig-Holstein, Campus Lübeck, 23538 Lübeck, Germany; 4 Division of Physiological Chemistry I, Department of Medical Biochemistry and Biophysics, Karolinska Institutet, 171 77 Stockholm, Sweden; 5 Proteomics Biomedicum, Division of Physiological Chemistry I, Department of Medical Biochemistry and Biophysics, Karolinska Institutet, 171 77 Stockholm, Sweden; 6 Department of Oncology Pathology, Karolinska Institutet, 171 64 Solna, Sweden

**Keywords:** Colorectal cancer, Metastasis, Panomics, Tumour heterogeneity, Patient individuality, Biomarkers

## Abstract

**Background:**

Colorectal cancer (CRC) is one of the most prevalent cancers, with over one million new cases per year. Overall, prognosis of CRC largely depends on the disease stage and metastatic status. As precision oncology for patients with CRC continues to improve, this study aimed to integrate genomic, transcriptomic, and proteomic analyses to identify significant differences in expression during CRC progression using a unique set of paired patient samples while considering tumour heterogeneity.

**Methods:**

We analysed fresh-frozen tissue samples prepared under strict cryogenic conditions of matched healthy colon mucosa, colorectal carcinoma, and liver metastasis from the same patients. Somatic mutations of known cancer-related genes were analysed using Illumina's TruSeq Amplicon Cancer Panel; the transcriptome was assessed comprehensively using Clariom D microarrays. The global proteome was evaluated by liquid chromatography-coupled mass spectrometry (LC‒MS/MS) and validated by two-dimensional difference in-gel electrophoresis. Subsequent unsupervised principal component clustering, statistical comparisons, and gene set enrichment analyses were calculated based on differential expression results.

**Results:**

Although panomics revealed low RNA and protein expression of CA1, CLCA1, MATN2, AHCYL2, and FCGBP in malignant tissues compared to healthy colon mucosa, no differentially expressed RNA or protein targets were detected between tumour and metastatic tissues. Subsequent intra-patient comparisons revealed highly specific expression differences (e.g., SRSF3, OLFM4, and CEACAM5) associated with patient-specific transcriptomes and proteomes.

**Conclusion:**

Our research results highlight the importance of inter- and intra-tumour heterogeneity as well as individual, patient-paired evaluations for clinical studies. In addition to changes among groups reflecting CRC progression, we identified significant expression differences between normal colon mucosa, primary tumour, and liver metastasis samples from individuals, which might accelerate implementation of precision oncology in the future.

**Supplementary Information:**

The online version contains supplementary material available at 10.1186/s12967-022-03855-0.

## Introduction

Colorectal cancer (CRC) is the third most common cancer and cause of cancer-related death [1]. Although early-stage CRC has a relatively good prognosis, with a 5-year survival of almost 90%, prognosis of metastatic CRC (mCRC) is poor, with a 5-year survival of only 15% [2]. In this context, over 50% of CRC patients either present with liver metastasis at the primary diagnosis or develop progression shortly thereafter [3, 4]. Furthermore, knowledge about disease progression and how tumour heterogeneity contributes to metastasis or treatment tolerance remains limited [5].

To understand the underlying molecular changes that drive the carcinogenesis of mCRC, The Cancer Genome Atlas (TCGA) has conducted extensive genomic, epigenomic, and transcriptomic profiling studies to identify distinguishing features [6–9]. These studies have underscored the value of molecular characterization in addition to histological assessment for stratification of mCRC patients while identifying genomic features unique to mCRC tumorigenesis [10]. Although it has been shown that the mutation status of specific oncogenes such as *HER2* in breast cancers, *EGFR* in lung cancers, and *KRAS* in colon cancers are crucial for targeted treatment [11, 12], genomic markers fail to identify eligible patients or predict therapy outcomes in the majority of cases. For patients with advanced or metastatic cancer, Marquart and colleagues estimated in 2018 that only 8.3% would be eligible for genome-driven drugs and that only 4.9% would benefit from these drugs [13]. In general, combining proteomics and genomics to proteogenomic analyses might improve individualized cancer medicine [14].

The proteome represents a central position: while it is regulated downstream by genetic expression and reacts to signals from the environment and treatments, the main task of proteins is to mediate the biochemical activities of cells and organs [15]. As such, proteins can help to determine the significance of a physiological phenotype and a point of intervention for drug and health treatments [16–18]. Indeed, several preclinical studies have identified the CRC proteome to explain the biological changes that affect this cancer. Interestingly, most proteomic studies to date have focused on differentially expressed proteins, though the patients used as the source of primary colorectal tumour samples and those for liver metastasis samples differed. For instance, Li et al. identified in 2020 metastasis-related factors that were differentially expressed in synchronous solitary liver metastasis compared to primary colon cancer [19]. By integrating the genomics, proteomics, and phosphoproteomics of 480 clinical tissues, molecular signatures are used to characterize three CRC subtypes. The authors detected high similarities with primary tumours at genetic but not proteomic levels. Similarly, Sardo et al. recently reviewed panomics approaches in CRC beyond genomic data, e.g., predictive proteomic targets in clinical settings [20]. However, little is currently known about panomics applied to individual-matched patient samples.

The purpose of this study was to perform an integrated panomics analysis of genomics, transcriptomics, and proteomics in mCRC by using paired clinical samples that characterize intra- and inter-tumour heterogeneity during tumour progression.

## Materials and methods

### Overview of the patient cohort

Paired tissue samples from normal adjacent colon mucosa (NM), corresponding primary colorectal carcinoma (T), and corresponding liver metastasis (LM) were obtained from four patients (P1-4). For one patient, we collected eight samples: normal colon mucosa, six samples from different locations within the primary tumour, and one sample from the corresponding liver metastasis. Table 1 presents all patient characteristics. All patients were diagnosed with metastasized colorectal carcinoma and underwent primary resection. Samples were surgically removed at the Department of Surgery, University Hospital Schleswig–Holstein, Campus Lübeck, and stored in liquid nitrogen until processing. All patients provided informed written consent. The Ethics Committee of the University of Lübeck gave ethical approval for this work (No. 07-124 and 16-282).

**Table 1:** Clinical parameters of the patient cohort

Patient	Sex	Age range (years)^a^	Staging (TNM)	Grading	Location of the primary tumour	Overall survival (months)	Months between resection of the primary tumour and liver metastasis
1	f	51–55	pT4 pN1 M1	G3	Ascending colon	28	1.2
2	m	41–45	pT1 pN0 M1	G2	Sigmoid colon	79	36.4
3	f	61–65	pT4a pN2a M1	G2	Coecum	16	0
4	m	71–75	pT3 pN1b M1	G2	Sigmoid colon	> 96	1.3

f, female; m, male; TNM, Tumour Node Metastasis Classification of Malignant Tumours

^a^The exact age of individual patients was removed to avoid patient identification

### Sample preparation concerning intra-tumour heterogeneity

All samples were manually divided into halves on dryice pre-chilled plates to allow for subsequent downstream analysis of intra-tumour heterogeneity while maintaining low temperature. Six-micron sections from both sides of the two halves were cut and stained with haematoxylin and eosin (H&E) for histopathologic classification. Semi-automated frozen aliquots (1.5 mm core) were subsequently taken from the areas with the highest tumour cell (or mucosal cell) representativity by using a CryoXtract CXT350 (CryoXtract Instruments, USA) at a temperature below − 100 °C; the samples were added to 700 µl lysis buffer (Qiagen buffer RLT plus, 1% β-mercaptoethanol). Additional data show representative images before and after the coring process (Additional file 1: Fig. S1).

For extraction of nucleic acids and proteins, AllPrep^®^ DNA/RNA Micro Kit (Qiagen, Germany) was used according to the manufacturer’s instructions with additional steps for AllPrep^®^ DNA/RNA/Protein Mini Kit (Qiagen, Germany) and PureLink™ DNase (Invitrogen, USA). This extraction protocol resulted in one 100 µl DNA, 60 µl RNA, and 10 µl miRNA solution as well as in a protein pellet dissolved in 200 µl lysis buffer. One hundred microlitres of each protein sample was purified with ReadyPrep 2-D Cleanup Kit (Bio-Rad, USA). The purified protein pellet was dissolved in 22 µl DIGE buffer [30 mM TRIS, 7 M urea, 2 M thiourea, 4% (w/v) CHAPS]. A 2 µl aliquot was used to determine total protein concentration with EZQ™ Protein Quantitation Kit (Life Technologies, USA). The remaining protein solution was stored at − 80 °C until analysis.

### Targeted detection of somatic mutations in cancer-related genes

TruSeq™ Amplicon Cancer Panel (TSACP, Illumina, San Diego, USA) is a multiplexed targeted amplicon sequencing (tNGS) assay to detect somatic mutations frequently reported as related to cancer. Library preparation was carried out according to Illumina’s TSACP standard protocol. All samples were sequenced in two runs with the MiSeq™ next-generation sequencing (NGS) system. To this end, the MiSeq Reagent Kit V2 was utilized at 300 cycles.

Several analysis procedures, including demultiplexing and FASTQ file generation, were performed using MiSeq reporter software (Real-Time-Analysis (RTA), Version: 1.18.54). Starting with FASTQ files containing raw paired-end data, an in-house software pipeline was applied for data analysis. Briefly, reads were mapped to the reference genome (GRCH38/hg38) with Burrows‒Wheeler Aligner (BWA-MEM v.0.7.17-4). PICARD TOOLS (v.2.3.2-1) was employed to sort the resulting SAM files and for conversion to the BAM format. Adjustment of quality scores was carried out using the Base Quality Score Recalibration (BQSR) method provided by GATK (v. 4.2.6.1). The preprocessed BAM files are input for several working steps that are provided by tools of the GATK best practices workflow (GATK v. 4.2.6.1) for detection of somatic short variants [single-nucleotide variants (SNVs) and insertions/deletions (Indels)]. This workflow includes the use of Mutect2 for computation of a basic callset of candidate variants and subsequent filtering with the FilterMutectCalls algorithm of GATK. Ensembl Variant Effect Predictor (VEP, release 106) was applied to annotate the filtered variants with information regarding the effects of the somatic variants detected. Next, annotations (in mutation annotation format, MAF) were imported into R (v. 4.2.0). Several cleaning/filtering procedures were performed (e.g., entries in the call set not mapping to genes included in TSCAP, variants with low coverage or variants with population allele frequency > 0.001 in the gnomAD or 1k genome databases were removed). The filtered data were set as the input for the R package maftools (package version 2.12.0) for data visualization and computation of summary metrics for the data set [21].

### Transcriptome profiling

Data generation was performed according to the vendor’s original protocol using GeneChip™ WT Pico Reagent Kit (Thermo Scientific, USA), followed by hybridization with the Clariom D array (Clariom™ D Pico Assay, human, Thermo Scientific). Clariom D array data were imported into R (v4.1.2) using the oligo package (v1.60.0) preprocessed utilizing the robust multichip average algorithm (RMA). Annotations were added using the *annotateEset* function implemented in the affycoretools (v1.68.0) package and clariomdhumantranscriptcluster.db (v8.8.0) as annotations. Probes not matching known genes were removed before analysis.

### Mass spectrometry profiling

Equal aliquots of 47 µg of protein from each protein sample were diluted with DIGE buffer [30 mM TRIS, 7 M urea, 2 M thiourea, 4% (w/v) CHAPS] to a total volume of 50 µl and cleaned with a filter-aided sample preparation protocol (FASP) [22]. Dried protein pellets were diluted in 40 µl of 5% formic acid before mass spectrometric analysis, and half of the sample was prepared on a StageTip as previously described [23]. Peptides were separated chromatographically using a 25 cm-long C18 column (SilicaTip™ 360 µm OD, 100 µm ID, New Objective, USA) and an EASY-nLC1000™ nanoflow LC system (Thermo Fisher Scientific, USA). With a 300 nl/min flow rate, peptides were eluted at a linear gradient from 2 to 26% solvent B (0.1% formic acid in 98% acetonitrile) for 120 min. Mass spectrometric detection of the eluted peptides was carried out using a Q Exactive™ Plus hybrid quadrupole-Orbitrap™ mass spectrometer (Thermo Fisher Scientific, Germany) in data-dependent mode. The survey mass spectrum was acquired at a resolution of 140,000 (at *m/z* 200) in the range of *m/z* 300–1650, targeting 5 × 10^6^ ions. The MS/MS data for the 16 most intense precursors were obtained with higher-energy collisional dissociation (HCD) set at 28% normalized collision energy following isolation of precursor ions with 4Th targeting 2 × 10^5^ ions with charge z > 1 at a resolution of 17,500.

Tandem mass spectra were extracted using Raw2MGF (in-house program), and the resulting Mascot generic files (.mgf) were searched against a concatenated SwissProt protein database (Human taxonomy) using the Mascot 2.3.0 search engine (Matrix Science Ltd., UK). Carbamidomethylation of cysteines was set as a fixed modification, and deamidation of asparagine and glutamine as well as oxidation of methionine were set as variable modifications. Up to two missed tryptic cleavages were allowed, and the mass tolerance was set to 10 ppm and 0.05 Da for precursor and fragment ions, respectively. Only peptides with individual MS/MS Mascot scores above the significant threshold of E < 0.05 were accepted. Only proteins identified with at least two peptides with a significance score and a 0.25% false discovery rate (FDR) were considered for further quantification.

The mass spectra acquired were analysed with in-house-developed Quanti software (v2.5.4.4) using the relative abundance of proteins identified with more than two unique peptides [24]. The minimal requirements were a peptide length of six amino acids and FDR of 0.01. The areas of chromatographic peaks were taken as peptide abundances, and the same peptides were quantified in each nano-LC‒MS/MS data file using accurate mass and the order of elution as identifiers. The following settings were applied: (1) enzyme "trypsin", (2) fixed modification "cysteine carbamidomethyl", (3) optional modifications "methionine oxidation, asparagine, and glutamine deamidation, N-terminal acetylation", and (4) a maximum of two missed cleavages. The results were analysed in the R scripting and statistical environment. Data were normalized by calculating the summed intensities of all proteins in each sample and the median of all these summed intensities over the entire sample set. Each quantitative value was multiplied by the median/summed intensity, and the resulting values were log2 transformed. Differences in relative protein abundances between the treatment and control samples were assessed by a moderated t-test using the limma package [25]. Benjamini‒Hochberg correction was applied for multiple comparisons.

### Protein profiling by two-dimensional gel electrophoresis

Paired patient clustering was validated by multiplex fluorescent two-dimensional gel electrophoresis (2-D DIGE). Refraction-2D™ Labelling Kit (NH DyeAGNOSTICS, Germany) was used to label 50 µg of each protein sample, a pool of the tumour samples from P3, and an internal standard described previously [26]. Briefly, proteins were applied to an immobilized pH gradient gel strip (pH range 4–7, GE Healthcare, UK) for active rehydration and separated by SDS‒PAGE using precast 12.5% acrylamide gels (Bio-Rad, USA). Gel images were acquired with a Typhoon FLA 9000 scanner (GE Healthcare, UK) and analysed with Progenesis SameSpots (Nonlinear Dynamics, USA; v4.5). Spots were aligned to a reference image, automatically detected, manually corrected, and normalized to the internal standard.

### Statistical analysis

LC‒MS/MS data were analysed using the R statistical environment. Proteins with missing values in at least one sample and proteins with unknown gene names were removed. The remaining data were normalized using quantile normalization from the preprocessCore R package (v1.52.1).

To cluster samples based on RNA and protein expression profiles, principal component analysis was performed using the 100 proteins with the highest variance (FactoMineR R package, v2.4, [27]). Differentially abundant genes and proteins were detected using a linear model approach from the limma package for R (v3.46.0) [25]. Two-group comparisons of NM vs. T, NM vs. LM, and T vs. LM were carried out across all patients. The within-patient correlation was estimated using the duplicateCorrelation function Field [27] to correct repeated measurements in the same patients. A significance level of q < 0.01 and a relevant fold change (FC) of |log_2_FC| > 1 were applied for two-group comparisons. Individual patient comparisons were evaluated by calculating the correlation factor ρ and plotting the protein FCs for NM vs. T and NM vs. LM against each other. Due to the more significant difference between individual patient comparisons, the effect size was set to |log_2_FC| > 2.

Identified proteins in each sample were further enriched against HALLMARK gene sets (MSigDB v7.1) using Generally Applicable Gene Set Enrichment (GAGE), as implemented in the R package gage (v2.40.1) [28, 29].

2-D DIGE was analysed by SameSpots software (v4.5, Nonlinear Dynamics, USA). For ANOVA two-group comparisons, a p-value < 0.05 and |log_2_FC| > 1 were considered significant.

### Data availability

The transcriptomic expression data are available at Gene Expression Omnibus (GEO, https://www.ncbi.nlm.nih.gov/geo/) under accession number GSE206800. The mass spectrometry proteomics data have been deposited at ProteomeXchange Consortium via the PRIDE partner [30] repository with the data set identifier PXD036434.

## Results

To compare differences between fresh frozen samples obtained from patients with advanced-stage CRC, we performed panomics by TruSeq™ Amplicon sequencing, Clariom D arrays, quantitative mass spectrometric profiling, and 2-dimensional gel electrophoresis. For all patients, primary tumour tissue was collected before chemotherapy. All patients had synchronous liver metastasis at the time of diagnosis (Table 1), and except for one patient (P2), all metastatic tissues were taken before exposure to chemotherapy. For P4, screening six different primary tumour locations was also possible.

### Characterization of paired patient samples using gene expression data and mutation analysis

To identify genomic features, we analysed mutational data of the selected cohort (targeted sequencing). Frequently mutated genes were *APC* (100%), *GNA11* (100%), *TP53* (100%), *ERBB2* (75%), *KRAS* (62%), and *ATM* (62%). All 24 genes with a detectable mutation are presented in Additional file 1: Fig. S2. We did not observe any significantly unbalanced distribution between malignant groups and individual patients when considering the mutated genes.

**Fig. 1:**
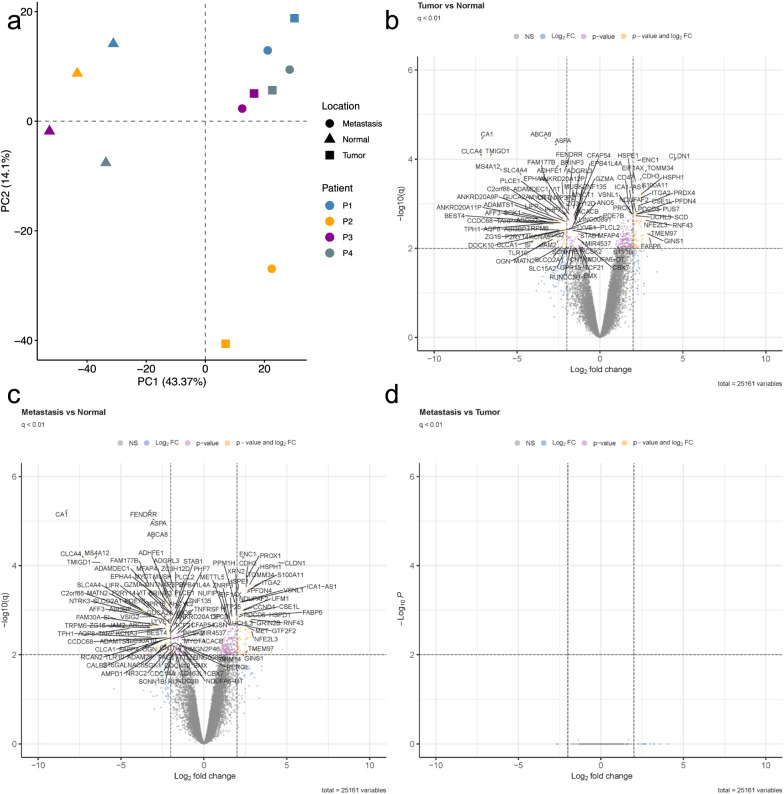
Unsupervised principal component analysis (**a**) and volcano plots of differentially expressed genes between all three group comparisons for NM, T, and LM (**b**–**d**). The PCA plot displays all four individual patients (P1, blue; P2, yellow; P3, purple; P4, grey). The X- and y-axes show the first and second principal components, respectively. Volcano plots are presented with the fold-change of the corresponding comparison in logarithmic scale (x-axis) against the q-value (y-axis) of **b** tumour vs. normal mucosa, **c** metastasis vs. normal mucosa, and **d** metastasis vs. tumour comparisons. Significance thresholds (q-value < 0.01 and |log_2_FC| threshold of > 2) are indicated by dashed lines. Genes passing these cut-offs were considered significant and coloured in yellow. Genes passing the q-value but not the log_2_FC threshold are coloured in rose. Genes that were not significant but passed the log_2_FC threshold are indicated in blue

Gene expression profiles for 25,161 genes were retrieved from microarrays and visualized using the top 2000 most variable genes (Fig. 1a). Remarkably, the patient and non-disease groups clustered together. In total, 130 genes were identified as significantly differentially expressed between NM and corresponding T samples (q-value < 0.01; |log_2_ FC| > 2; 62 more highly expressed in NM, 68 more highly expressed in T; Fig. 1b) and 154 genes between NM and corresponding LM (81 more highly expressed in NM, 73 more highly expressed in LM, Fig. 1c). Conversely, no differences in gene expression between LM and T were detected (Fig. 1d).

### Proteomic characterization of paired patient samples

Using a robust label-free workflow, all samples showed a high proteome depth and were included in subsequent analysis (Additional file 1: Fig. S3).

**Fig. 2:**
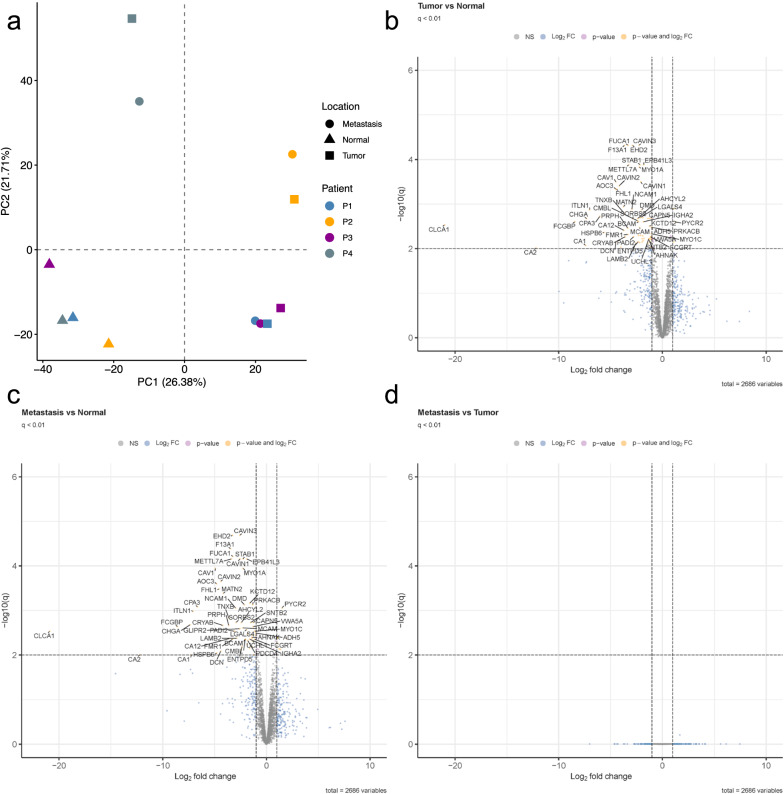
Unsupervised principal component analysis (**a**) and volcano plots of differentially expressed proteins between all three group comparisons for NM, T, and LM (**b**–**d**). The PCA plot displays all four individual patients (P1, blue; P2, yellow; P3, purple; P4, grey). The X- and y-axes show the first and second principal components, respectively. Volcano plots are presented with the fold-change of the corresponding comparison in logarithmic scale (x-axis) against the q-value (y-axis) of **b** tumour vs. normal mucosa, **c** metastasis vs. normal mucosa, and **d** metastasis vs. tumour comparisons. Significance thresholds (q-value < 0.01 and |log_2_FC| threshold of > 2) are indicated by dashed lines. Proteins passing these cut-offs were considered significant and coloured in yellow. Proteins passing the q-value but not the log_2_FC threshold are coloured in rose. Proteins that were not significant but passed the log_2_FC cut-off are indicated in blue

A total of 2885 proteins were identified by LC‒MS/MS. After removing missing values, unsupervised clustering using PCA for 2686 protein groups revealed one close cluster of all NM samples (Fig. 2a). In line with the transcriptomics data, patients (P1-P4) and not disease groups (T, LM) presented distinct similarities pointing to individual clinical phenotypes. Comparison between NM and T samples yielded 71 significantly differentially expressed proteins (q-value < 0.01 and |log_2_ FC| > 1), with 68 being (96%) up-regulated and three (4%) down-regulated in NM samples (Fig. 2b). Similarly, in NM and LM comparison, 69 proteins were significantly differentially expressed (q-value < 0.01 and |log_2_ FC| > 1): 61 (88%) up-regulated and eight (12%) down-regulated in NM (Fig. 2c). The chloride channel accessory 1 protein (CLCA1) was detected at an exceptionally significant low level in both T and LM compared to NM (log_2_FC_TvsNM_ = − 21.010 and log_2_FC_LMvsNM_ = − 20.888).

The most surprising aspect of the data was that no protein could be detected as being significantly expressed between T and LM, highlighting inter-patient heterogeneity (Fig. 2d).

### Comparison of gene and protein expression data and validation of the global proteome by 2-D DIGE

**Fig. 3:**
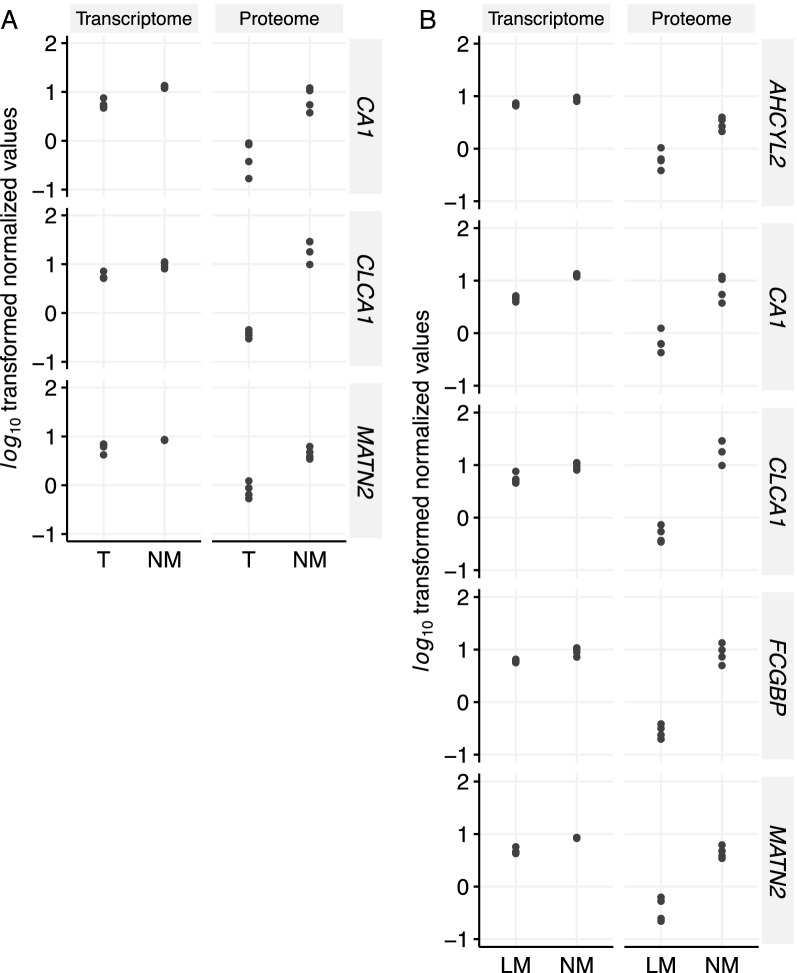
Overlap of the transcriptome and proteome data showing significantly expressed targets for NM vs. T (**a**) and NM vs. LM (**b**) group comparisons. NM, normal mucosa; T, tumour; LM, liver metastasis

Closer inspection of the gene (n = 25,161) and protein (n = 2686) expression data showed an overlap of three genes/proteins for the NM vs. T comparison (CA1, CLCA1, MATN2) and five for the NM vs. LM comparison (AHCYL2, CA1, CLCA1, FCGBP, MATN2) that were significantly expressed between the groups. All genes/proteins were characterized by higher levels in normal material, as plotted based on gene and protein abundances (Fig. 3). In agreement, increased expression of all targeted genes were associated with good prognosis in CRC when using data from Human Protein Atlas [31, 32].

Two-dimensional gel electrophoresis was performed to validate the clustering results for the proteome profiling. SameSpots software detected 1334 spots per gel. PCA based on these results indicated a qualitatively similar result, suggesting a clear distinction between patients and sample groups (Additional file 1: Fig. S4).

### The individual progression proteomic landscape in individual patients

Based on the group comparison results, each primary tumour's transcriptome and proteome were compared with paired adjacent mucosa and associated liver metastasis. All tumour samples correlated phenotypically closely with their corresponding liver metastases but presented different gene/protein profiles compared to their adjacent normal mucosa. The Pearson correlation for gene expression and protein abundance between the primary tumours and paired liver metastases compared to their adjacent mucosa was 0.6854 < r < 0.8281 for the proteome and 0.6114 < r < 0.8133 for the transcriptome (Additional file 1: Figs. S5–S8).

### Patient 1: female, right-sided CRC (pT4)

Proteome analysis of NM1, T1, and LM1 revealed proteome expression changes between the initial diagnosis and surgery for metastasis after one month. Of the 2,686 evaluable proteins, 294 showed |log_2_ FC| > 2 in the NM1 vs. T1 comparison and 251 in the NM1 vs. LM1 comparison (Additional file 1: Fig. S5a). The comparison between T1 and LM1 revealed 55 contrasting proteins (Additional file 1: Fig. S5b). Additional file 2: Table S1 lists the top differentially expressed proteins of the three two-group comparisons. Among proteins with higher abundance in LM1 than T1, CD74 (log_2_ FC = 2.179) is reported to be an oncogene (Network of Cancer Genes 7.0, http://ncg.kcl.ac.uk) that promotes tumour growth and metastasis in various cancer types. Tenascin C (TNC), the second detectable oncogene, was more highly expressed in T1 (log_2_ FC = 6.522) and LM1 (log_2_ FC = 2.930) than in NM1 but showed lower levels in LM1 than in T1 (log_2_ FC = − 3.592).

At the RNA level, 30 transcripts in the NM1 vs. T1 comparison and 34 in the NM1 vs. LM1 comparison were differentially expressed at |log_2_ FC| > 1 (Additional file 1: Fig. S5c). Contrasting RNAs were found in the T1 vs. LM1 comparison, with no genes showing |log_2_ FC| > 1 (Additional file 1: Fig. S5d). Additional file 2: Table S2 lists the top differentially expressed RNAs with either higher or lower gene expression for each two-group comparison. Among RNAs with a higher level in LM1 than T1, *SFRP4* was previously reported as an oncogene (Network of Cancer Genes 7.0).

The overlap of differentially expressed genes and proteins showed three molecules for the NM1 vs. T1 comparison (CA1, CLCA1, ZG16) and six for the NM1 vs. LM1 comparison (CA1, CLCA1, FABP4, MUC2, PIGR, ZG16). The abundance of CEACAM5, which has been widely applied in clinical detection of liver metastasis of CRC, showed almost no differential expression between NM1, T1, and LM1 at RNA and protein levels (Additional file 1: Fig. S5).

### Patient 2: male, left-sided CRC (pT1)

Proteome analysis of samples from another patient (NM2/T2 and LM2) indicated proteome expression changes between initial diagnosis and metastasis surgery after 36 months. The patient underwent several chemotherapies during that timeframe. In total, 183 proteins in the NM2 vs. T2 comparison and 265 in the NM2 vs. LM2 comparison were differentially expressed (|log_2_ FC| > 2, Additional file 1: Fig. S6a and b). The comparison between T2 and LM2 revealed only 70 contrasting proteins. Additional file 2: Table S3 provides the top differentially expressed proteins with either a higher or lower protein concentration for each comparison. Among proteins with a higher level in LM2 than T2, SRSF3 (Serine and Arginine Rich Splicing Factor, log_2_ FC = 2.013) is the only reported oncogene (Network of Cancer Genes 7.0). Interestingly, SRSF3 was also strongly more highly expressed in T2 (log_2_ FC = 2.944) and LM2 (log_2_ FC = 4.956) than in NM2. CEACAM5, a potential marker for CRC progression, was also strongly upregulated in T2 and LM2 compared to NM2.

Regarding gene expression data, 53 RNAs revealed |log_2_ FC| > 1 in the NM2 vs. T2 comparison and 59 in the NM2 vs. LM2 comparison. The comparison of T2 vs. LM2 revealed 13 differentially expressed RNAs (Additional file 1: Fig. S6c and d). The top differentially expressed RNAs of the three two-group comparisons are presented in Additional file 2: Table S4. The overlap of differentially expressed genes and proteins showed nine features for the NM2 vs. T2 comparison (CA1, CLCA1, CPA3, EPB41L3, JCHAIN, MATN2, MUC2, OGN, SULT1B1) and eight for the NM2 vs. LM2 comparison (CA1, CLCA1, MATN2, MEP1A, MUC2, PIGR, SULT1B1, ZG16). PIGR is an annotated healthy driver (Network of Cancer Genes 7.0) with lower RNA (log_2_ FC = − 1.165) and protein (log_2_ FC = − 2.676) levels in LM2 than NM2.

### Patient 3: male, left-sided CRC (pT3)

Proteome analysis of the samples from a third patient (NM3/T3 and LM3) indicated proteome expression changes between initial diagnosis and metastasis surgery after one month. In total, 328 proteins in the NM3 vs. T3 comparison and 264 in the NM3 vs. LM3 comparison were differentially expressed (|log_2_ FC| > 2, Additional file 1: Fig. S7a and b). In total, 83 contrasting proteins were obtained through the comparison between T3 and LM3. Additional file 2: Table S5 gives the top 15 differentially expressed proteins (low & high), including the two strongly expressed oncogenes, i.e., CD74 and TNC, and the clinically applicable biomarker for CRC liver metastasis CEACAM5.

Furthermore, 19 RNAs in the NM3 vs. T3 comparison and 35 in the NM3 vs. LM3 comparison were differentially expressed (|log_2_ FC| > 1). The comparison between T3 and LM3 revealed only nine differentially expressed genes (the top genes are presented in Additional file 2: Table S6). Interestingly, only CA1 was found at a lower level in both malignant tissues than in adjacent mucosa at both RNA and protein levels (RNA level: log_2_ FC = − 1.208 and − 1.402; protein level: log_2_ FC = − 2.170 and − 3.133, Additional file 1: Fig. S7c and d).

### Patient 4: female, right-sided CRC (pT4)

Samples from the fourth patient were retrieved during one simultaneous surgery. This opened the possibility of not merely collecting one sample of the tumour and its paired liver metastasis but six distinct tumour partitions. Semi-quantitative protein profiling by LC‒MS/MS revealed 329 proteins to be differentially expressed in NM4 vs. T4 (pooled) and 249 proteins in NM4 vs. LM4 comparison (|log_2_ FC| > 2). Comparing T4 and LM4 showed 155 differentially expressed proteins (Additional file 1: Fig. S8a and b, top proteins are shown in Additional file 2: Table S7). Interestingly, almost all tumour-associated proteins, including the oncogenes *DEK* and *CHD4* and the tumour-suppressor gene *MYH9*, were expressed at lower levels in LM4 than in T4. In contrast, CEACAM5 was more highly expressed in LM4. Particular attention should be given to TNC, SRSF3, and OLFM4, which presented the highest protein expression in T4 and LM4 compared to NM4.

Based on transcriptomic analysis, 35 RNAs showed |log_2_ FC| > 1 in the NM4 vs. T4 comparison; 61 showed |log2 FC| > 1 in the NM4 vs. LM4 comparison. Comparing T4 vs. LM4 revealed only four differentially expressed targets (Additional file 1: Fig. S8c and d). Additional file 2: Table S8 lists the top differentially expressed RNAs for the three two-group comparisons. The overlap of genes and proteins showed two molecules for the NM4 vs. T4 comparison (OGN, ORM1), one for the NM4 vs. LM4 comparison (CA1), and one for the T4 vs. LM4 comparison (FGG).

### The intra-tumoral proteomic landscape in samples from patient 4

Six distinct parts of the primary tumour of P4 were analysed to assess how different tumour biopsies might display intra-tumoral heterogeneity at the transcriptome and proteome levels. Interestingly, the Pearson correlation between T4(1–6) vs. NM4 and LM4 vs. NM4 ranged from 0.3596 < r < 0.7032 at the protein level and from 0.2100 < r < 0.8043 at the transcriptome level (Additional file 1: Figs. S9 and S10), highlighting different RNA and protein compositions for the six biopsies. Frequently mutated genes were *ERBB2* (100%), *APC* (100%), *GNA11* (100%), *KRAS* (100%), *PIK3CA* (100%), *SMAD4* (100%), and *TP53* (100%)*.* All 17 genes with a detectable mutation are presented in Additional file 1: Fig. S11.

### Functional enrichment analysis of detected proteins

Gene set enrichment analysis using HALLMARK gene sets was performed to obtain more comprehensive insight into the biological and functional characteristics of cancer progression in individual patients. Because of the small effect size, RNA expression data were not used for functional enrichment. With the threshold of FDR < 0.05, the protein expression differences indicated distinct activated pathways during the normal-to-tumour-to-metastasis transition for individual patients.

**Fig. 4:**
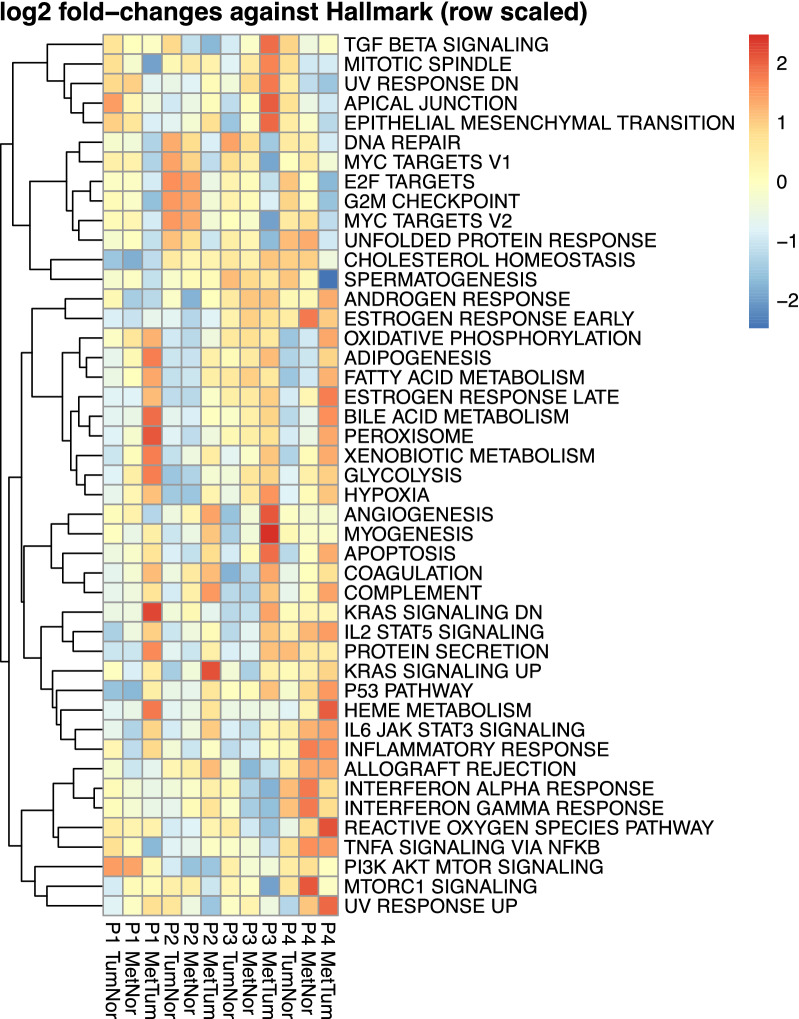
GAGE against HALLMARK gene sets using protein expression data for all two-group comparisons and all patients

Pathway analysis for P1 revealed xenobiotic metabolism, coagulation, and KRAS signalling as the top enriched hallmark gene sets in LM1 compared to NM1 and T; P2 showed activated pathways associated with angiogenesis, coagulation, and the complement system. Proteomic profiling for P3 resulted in activated pathways related to the epithelial-mesenchymal transition (EMT), myogenesis, and angiogenesis, P4 showed signatures correlating with oxidative phosphorylation, reactive oxygen species pathway, and bile acid metabolism (Fig. 4). The most striking results from the data are that: (i) individual pathways in a given patient is utilized during metastasis, and (ii) the most activated pathways were detected in T vs. LM comparisons.

## Discussion

In this report, we describe a panomics investigation of CRC that integrates a unique set of paired samples of the colon mucosa, primary tumours, and liver metastasis to provide insight into the impact of progression in individual patients. To ensure a robust model and patient comparison, we developed a new protocol to prepare fresh frozen samples with no freeze-and-thaw steps followed by proteomic approaches. This workflow robustly identified potential targets for early detection, diagnosis, and/or therapeutic intervention.

Our panomics approach indicated differentially expressed genes and proteins between adjacent normal mucosa and malignant tissue (T & LM, Figs. 1 and 2). As one of the few features overlapping between RNA and protein data in our group comparisons, CLCA1 was significantly lower expressed in malignant tissue than in healthy colonic mucosa (RNA: log_2_FC_TvsNM_ = − 4.02, q-value = 0.0068; log_2_FC_LMvsNM_ = − 4.12, q-value = 0.0057; protein: log_2_FC_TvsNM_ = − 21.01, q-value = 0.0029; protein log_2_FC_LMvsNM_ = − 20.88, q-value = 0.0029). This finding supports evidence from previous observations [33, 34] that show decreased expression of CLCA1 during CRC carcinogenesis. The CLCA1 protein, the gene for which is located at 1p22.3, is predominantly expressed in intestinal crypts and goblet cells of the colon mucosa. It activates calcium-dependent chloride channels and is primarily involved in mucus regulation [35, 36]. The tumour-suppressive function of CLCA1 is based on inhibition of EMT and the Wnt/β-catenin signalling pathway: both pathways reduce proliferation but promote differentiation, migration, and invasion [34, 37]. CLCA1 has already been identified as a prognostic factor in CRC, whereby a low expression level is associated with poor survival and advanced disease stages [38, 39]. Nevertheless, the exact mechanism of decreased expression of CLCA1 in malignant tissues needs to be elucidated in detail. Carbonic anhydrase-1 (CA1, 8q21.2) is reported to be decreased in CRC [40], which is consistent with our results (RNA: log_2_FC_TvsNM_ = − 4.02, q-value = 0.0068; log_2_FC_LMvsNM_ = − 4.12, q-value = 0.0057, protein: log_2_FC_TvsNM_ = − 21.01, q-value = 0.0029; log_2_FC_LMvsNM_ = − 20.88, q-value = 0.0029). CA1 belongs to the large family of zinc metalloenzymes, which maintain pH homeostasis [41] and catalyse the reversible hydration and dehydration reactions of CO_2_/H_2_CO_3_ [42]. Previous studies have shown that CA1 mRNA is significantly reduced in colon carcinoma and that loss of CA1 expression is associated with the disappearance of differentiated epithelial cells [43]. Accordingly, mRNA, RNA, and protein levels of CA1 are reduced in CRC according to data from Human Protein Atlas [31, 44].

Another important finding was the lower RNA- and protein level of matrilin-2 (MATN2) in tumour compared to normal tissues (RNA: log_2_FC_TvsNM_ = − 2.45, q-value = 0.0093; log_2_FCM_LMvsNM_ = − 3.71, q-value = 0.0010, protein: log_2_FC_TvsNM_ = − 3.75, q-value = 0.0011; log_2_FCM_LMvsNM_ = − 4.16, q-value = 0.0004). MATN2 is the largest member of the matrilin family, can bind to fibrillar collagens, and plays a role in cell growth and tissue remodelling [45, 46]. MATN2 expression has been shown to be significantly altered during cancer progression [47–49].

Most strikingly, we detected patient individuality and not group affiliation as the primary driver of the transcriptome and proteome in CRC. Each primary carcinoma was closely associated with its metastatic counterpart using cluster algorithms, without showing drastic mutational differences (Figs. 1a and 2a). First, these results are consistent with other studies reporting significant inter-individual heterogeneity in the clinicopathologic characteristics in CRC. For example, it has been demonstrated that, e.g., stratification along with right-sided or left-sided tumours, according to Consensus Molecular Subtypes by Guinney et al., impact prognosis [50]. Second, these findings highlight the individual evaluation of patients and comparison of entity groups. Most biomarker studies compare two or more groups that comprise specific disease subgroups and/or healthy individuals. However, by analysing paired patient samples, the expression data obtained in this investigation were more informative. Although we did not detect any differentially expressed genes and proteins between the T- and M-groups, the panomics landscape between individual patient T and M samples was drastically different (Figs. 1 and 2).

At the specific gene and protein levels, we found SRSF3, olfactomedin 4 (OLFM4), and the clinical routine marker CEACAM5 to be differentially expressed during patient-intra-individual CRC progression. SRSF3 was identified as a differentially expressed protein in P2 and P4, showing a high level in malignant tissues compared to adjacent normal mucosa (range log_2_FC_TvsNM_: 2.94–6.93; range log_2_FC_LMvsNM_: 4.93–4.96). SRSF3 (6p21.31-p21.2) is a target of the Wnt/β-catenin signalling pathway [51] and exerts its pro-oncogenic effects in different ways [52, 53]. SRSF3 is co-expressed with interleukin enhancer-binding factor 3 (ILF3) followed by increased alternative splicing of pro-proliferative ILF3 isoforms [54]; it also favours energy production via anaerobic glycolysis due to increased splicing of pyruvate kinase isoform M2 [55].

In addition to SRSF3, OLFM4 (13q14.3) showed a prominent expression increase in tumour and metastasis of P4 (log_2_ FC_T-NM_: 7.65; log_2_ FC_LM-NM_: 7.65), with no differences between tumour and metastasis samples (log_2_FC_LMvsT_: 0). OLFM4 is a glycoprotein involved in cell adhesion processes through cadherin interaction [56] and is predominantly expressed in crypts of the healthy colonic mucosa [57]. Our finding broadly supports other studies in this area linking OLFM4 over-expression with colorectal disease: van der Flier, Shinozaki, and Huang showed that higher protein levels of OLFM4 are associated with inflammatory bowel disease, adenomatous precursors, and CRC [57–59].

The detected protein levels of CEACAM5 reflect the challenge of inter- and intra-tumour heterogeneity and the importance of patient individuality. CEACAM5 (synonym for carcinoembryonic antigen, CEA) is over-expressed in approximately 90% of gastrointestinal, colorectal, and pancreatic cancers [60]. Serum levels of CEA are used clinically to monitor postoperative disease recurrence or response to cancer therapy in CRC, usually in combination with imaging and endoscopic procedures [61]. The current study found that CEACAM5 is heterogeneously expressed in primary and metastatic CRC tissue. Although a homogenous low level of CEACAM5 protein was detected in normal mucosa, protein expression increased heterogeneously per patient in malignant tissues. In detail, P1 and P4 presented an equal CEA level in both malignant tissues (T & LM), but CEA was increased or decreased in LMs of P2 and P3. This finding is consistent with other studies confirming strong intra- and inter-individual heterogeneity and limited diagnostic and prognostic value [62, 63].

Despite analysed a unique set of paired patient samples concerning tumour heterogeneity by integrating genomic, transcriptomic, and proteomic analyses, some limitations of the present study should be considered. First, markers detected may be reflective of therapeutic regimens after surgery for the primary tumour, which can affect the molecular composition of the disease during progression. Another potential limitation of this study was the variability of clinical parameters between individuals. Hence, the results of this small cohort may reflect bias associated with the collection of such clinical data. Therefore, validation of the changes among groups reflecting CRC progression requires future large-scale screenings.

## Conclusion

We showed that inter- and intra-tumour heterogeneity is clearly detectable by panomics and that patients and not disease groups present distinct similarities pointing to individual clinical phenotypes. In addition to changes among groups reflecting CRC progression (e.g., CA1, CLCA1, MATN2), we identified significant expression differences between individual patient tissues (e.g., SRSF3, OLFM4, CEACAM5), contributing to existing knowledge on patient-specific progression and highlighting the importance of paired samples for precision medicine. Additional studies using larger patient cohorts are needed to confirm our results and to investigate the biological function of the detected genes/proteins during CRC progression for novel patient stratification.

## Supplementary Information

**Additional file 1: Figure S1.** Representative images before and after coring. **Figure S2.** Oncoplot depicting 24 detectable mutated genes in P1-4 sorted and ordered by decreasing frequency. **Figure S3.** Number of proteins identified and quantified with a 1% FDR in each sample. **Figure S4.** Unsupervised principal component analysis of all three groups after two-dimensional gel electrophoresis. **Figure S5.** Panomics profiling of patient 1. Fold-change plots showing protein (a, b) and RNA (c, d) expression values for the NM1 vs T1 and NM1 vs LM1 (a, c) as well as for the T1 vs LM1 comparison (b, d). **Figure S6.** Panomics profiling of patient 2. Fold-change plots showing protein (a, b) and RNA (c, d) expression values for the NM2 vs T2 and NM2 vs LM2 (a, c) as well as for the T2 vs LM2 comparison (b, d). **Figure S7.** Panomics profiling of patient 3. Fold-change plots showing protein (a, b) and RNA (c, d) expression values for the NM3 vs T3 and NM3 vs LM3 (a, c) as well as for the T3 vs LM3 comparison (b, d). **Figure S8.** Panomics profiling of patient 4. Fold-change plots showing protein (a, b) and RNA (c, d) expression values for the NM4 vs T4 and NM4 vs LM4 (a, c) as well as for the T4 vs LM4 comparison (b, d). **Figure S9.** Protein profiling of patient 4 showing six different tumour locations. **Figure S10.** Transcriptomic profiling of patient 4 showing six different tumour locations. **Figure S11.** Oncoplot depicting 17 detectable mutated genes of different tumour locations in P4 sorted and ordered by decreasing frequency.

**Additional file 2: Table S1.** Top 30 differentially expressed proteins in two-group comparisons of patient 1. **Table S2.** Top 30 differentially expressed RNAs in two-group comparisons of patient 1. **Table S3.** Top 30 differentially expressed proteins in two-group comparisons of patient 2. **Table S4.** Top 30 differentially expressed RNAs in two-group comparisons of patient 2. **Table S5.** Top 30 differentially expressed proteins in two-group comparisons of patient 3. Listed are the top 15 proteins with increased or decreased expression in the comparison of tumour vs. normal tissue, metastasis vs. normal tissue, and metastasis vs. tumour tissue measured by log_2_FC. Proteins marked in bold are products of onco- or tumour suppressor genes annotated in NCG 7.0. **Table S6.** Top 30 differentially expressed RNAs in two-group comparisons of patient 3. Listed are the top 15 RNAs with increased or decreased expression in the comparison of tumour vs. normal tissue, metastasis vs. normal tissue, and metastasis vs. tumour tissue measured by log_2_FC. RNAs marked in bold are onco- or tumour suppressor genes annotated in NCG 7.0. **Table S7.** Top 30 differentially expressed proteins in two-group comparisons of patient 4. Listed are the top 15 proteins with increased or decreased expression in the comparison of tumour vs. normal tissue, metastasis vs. normal tissue, and metastasis vs. tumour tissue measured by log_2_FC. Proteins marked in bold are products of onco- or tumour suppressor genes annotated in NCG 7.0. **Table S8.** Top 30 differentially expressed RNAs in two-group comparisons of patient 4. Listed are the top 15 RNAs with increased or decreased expression in the comparison of tumour vs. normal tissue, metastasis vs. normal tissue, and metastasis vs. tumour tissue measured by log2FC. RNAs marked in bold are onco- or tumour suppressor genes annotated in NCG 7.0.

## Data Availability

Transcriptomic expression data are available at Gene Expression Omnibus (GEO, https://www.ncbi.nlm.nih.gov/geo/) with accession number GSE206800. The mass spectrometry proteomics data have been deposited at ProteomeXchange Consortium via the PRIDE partner repository with the data set identifier PXD036434.

## Abbreviations

2D-DIGE: Two-dimensional gel electrophoresis
CEA/CEACAM5: Carcinoembryonic antigen
CLCA1: Calcium-activated chloride channel regulator 1
CRC: Colorectal cancer
EMT: Epithelial-mesenchymal transition
FASP: Filter-aided sample preparation
GEO: Gene expression omnibus
H&E: Haematoxylin and eosin
HCD: High-energy collisional dissociation
LC‒MS/MS: Liquid chromatography-coupled mass spectrometry
LM: Liver metastasis
mCRC: Metastatic CRC
NGS: Next-generation sequencing
NM: Normal adjacent colon mucosa
OLFM4: Olfactomedin 4
RTA: Real-time analysis
SRSF3: Serine and arginine-rich splicing factor 3
T: Primary colorectal carcinoma
TCGA: The Cancer Genome Atlas
TNC: Tenascin C
tNGS: Targeted amplicon sequencing
TSACP: TruSeq™ Amplicon Cancer Panel
