# Supplementary material for: Panomics reveals patient individuality as the major driver of colorectal cancer progression
Source: J Transl Med. 2023 Jan 23;21:41. doi: 10.1186/s12967-022-03855-0 (PMC9869555; doi:10.1186/s12967-022-03855-0)
Supplement: Supplementary file 1 — Additional file 1: Figure S1. Representative images before and after coring. Figure S2. Oncoplot depicting 24 detectable mutated genes in P1-4 sorted and ordered by decreasing frequency. Figure S3. Number of proteins identified and quantified with a 1% FDR in each sample. Figure S4. Unsupervised principal component analysis of all three groups after two-dimensional gel electrophoresis. Figure S5. Panomics profiling of patient 1. Fold-change plots showing protein (a, b) and RNA (c, d) expression values for the NM1 vs T1 and NM1 vs LM1 (a, c) as well as for the T1 vs LM1 comparison (b, d). Figure S6. Panomics profiling of patient 2. Fold-change plots showing protein (a, b) and RNA (c, d) expression values for the NM2 vs T2 and NM2 vs LM2 (a, c) as well as for the T2 vs LM2 comparison (b, d). Figure S7. Panomics profiling of patient 3. Fold-change plots showing protein (a, b) and RNA (c, d) expression values for the NM3 vs T3 and NM3 vs LM3 (a, c) as well as for the T3 vs LM3 comparison (b, d). Figure S8. Panomics profiling of patient 4. Fold-change plots showing protein (a, b) and RNA (c, d) expression values for the NM4 vs T4 and NM4 vs LM4 (a, c) as well as for the T4 vs LM4 comparison (b, d). Figure S9. Protein profiling of patient 4 showing six different tumour locations. Figure S10. Transcriptomic profiling of patient 4 showing six different tumour locations. Figure S11. Oncoplot depicting 17 detectable mutated genes of different tumour locations in P4 sorted and ordered by decreasing frequency. [file 12967_2022_3855_MOESM1_ESM.docx]

**Figure S1: Representative images before and after coring**. Section of a tumour sample from P4 prior to coring, haematoxylin-eosin stained (A). The view direction from R3 is shown schematically. Section of the same sample after coring, haematoxylin-eosin stained (B). The circle with an inner diameter of 1.5 mm marks the punched area. While the dashed line outlines tumorous tissue (T), * marks connective tissue, and → a collection of lymphoid cells.


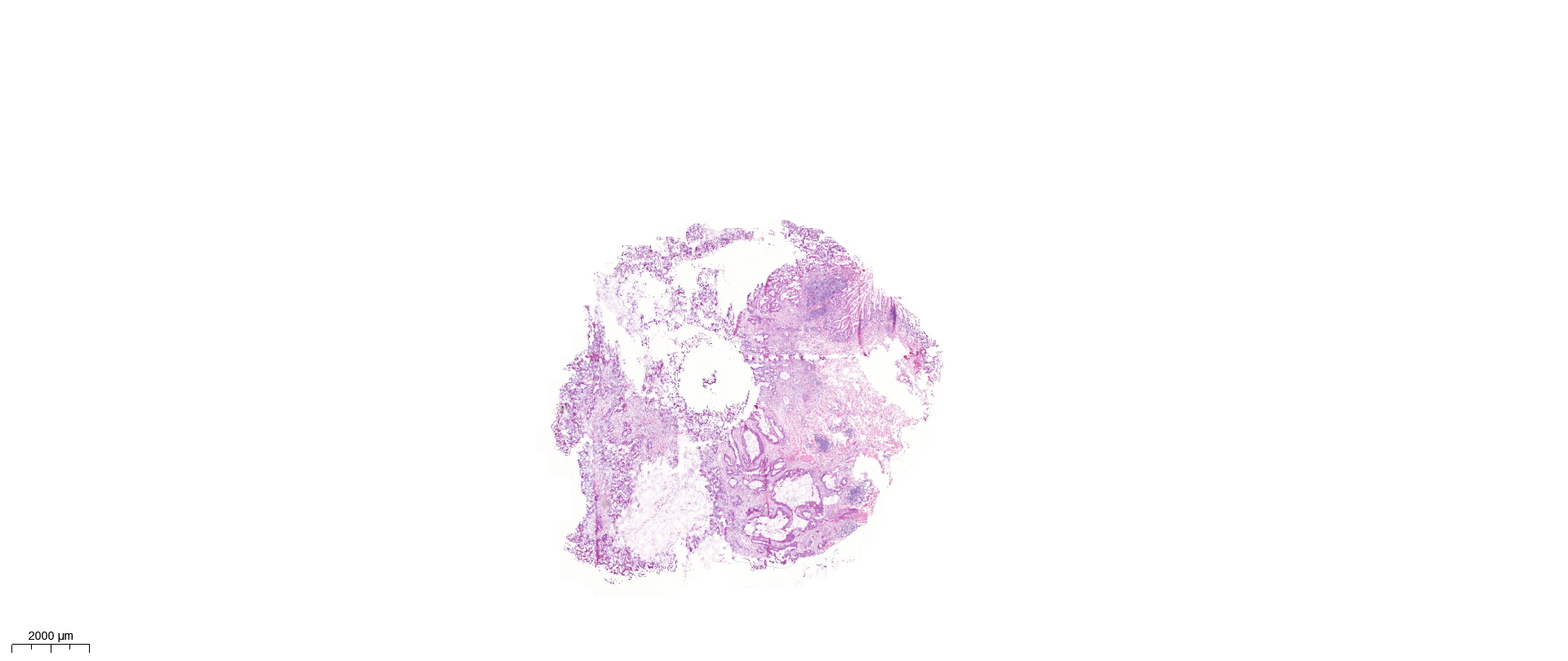

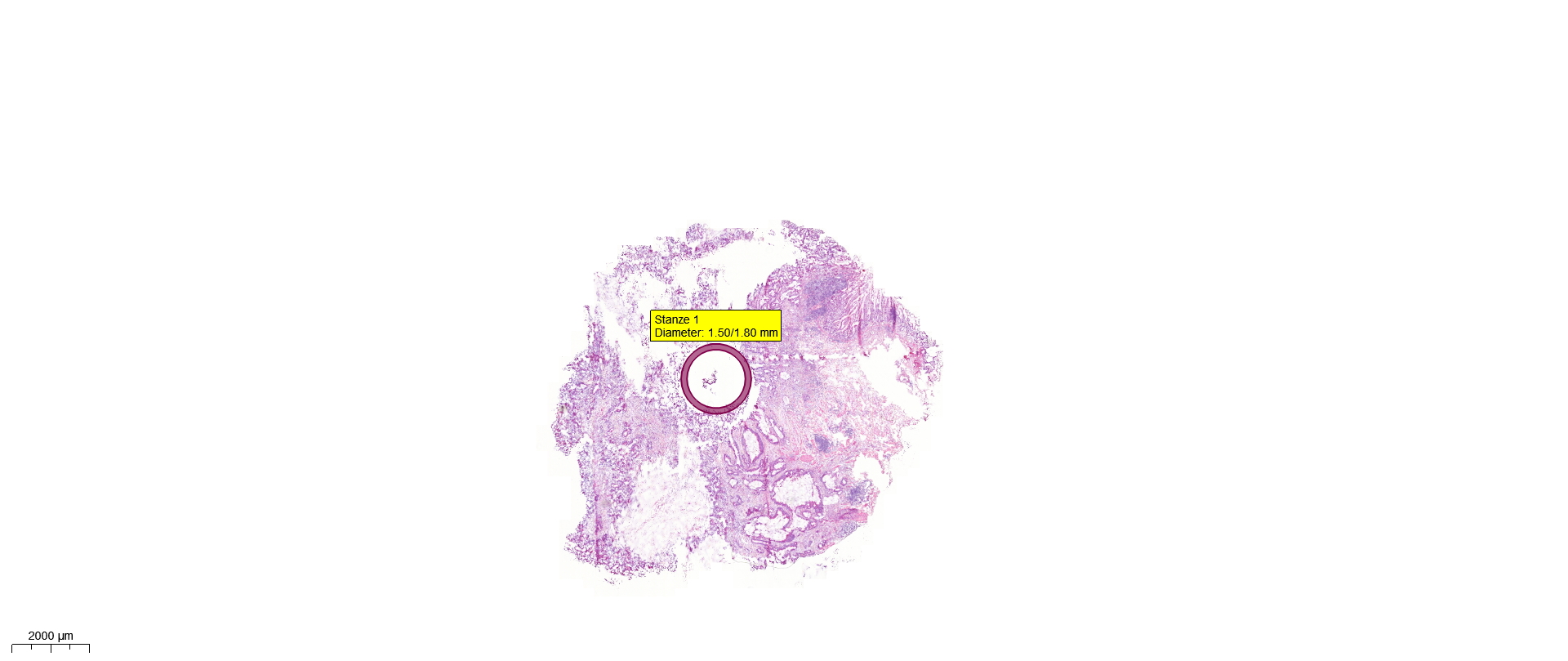

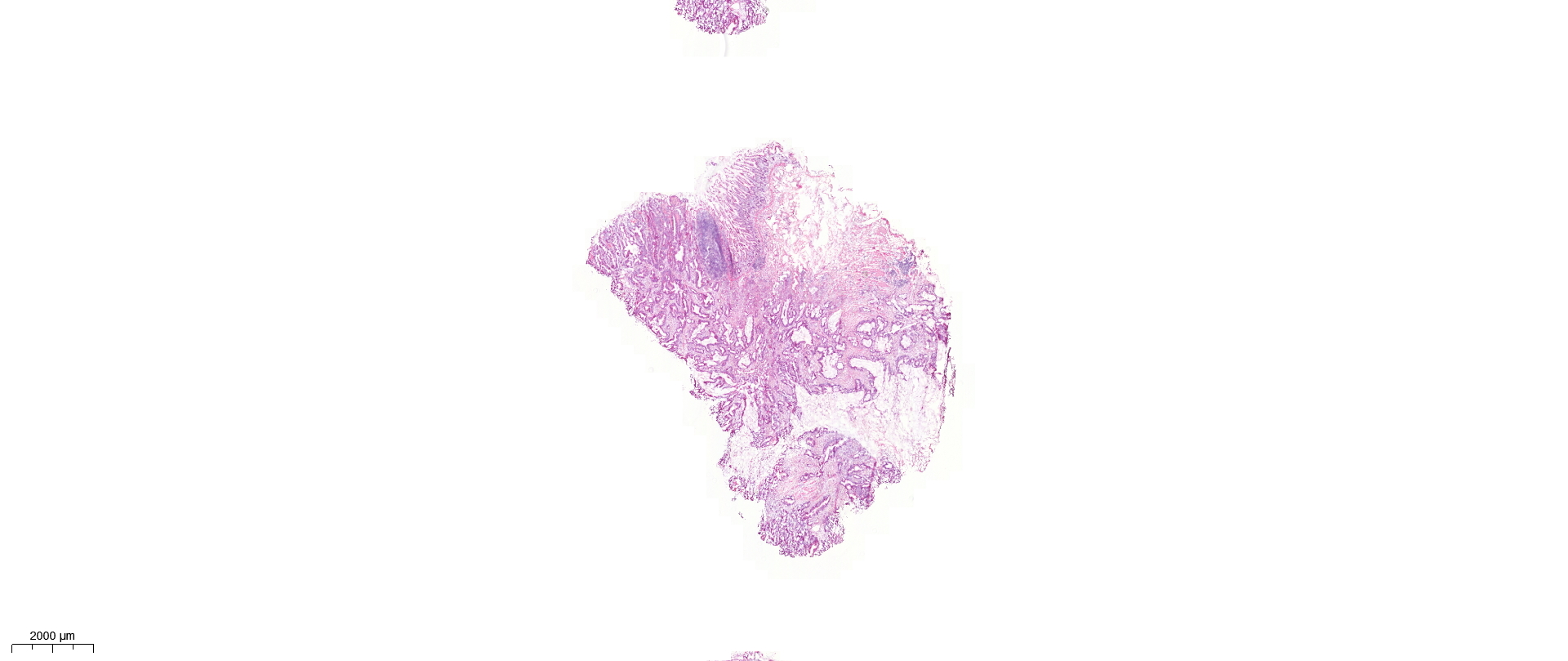


b

a


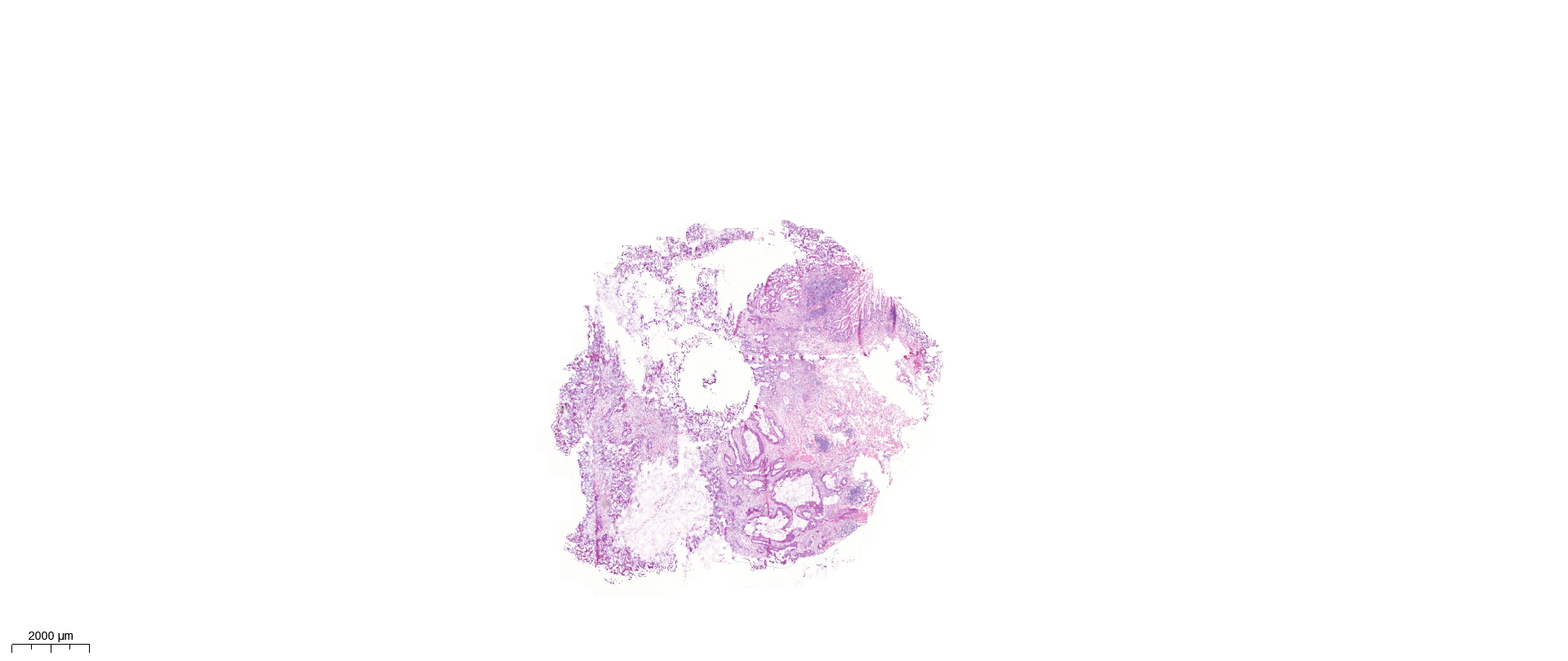


*

*

T

T

**Figure S2: Oncoplot depicting 24 detectable mutated genes in P1-4 sorted and ordered by decreasing frequency.** T, tumour; M, liver metastasis

******

**Figure S3: Number of proteins identified and quantified with a 1% FDR in each sample.** Bars indicate the mean and standard deviation. The complete dataset without any missing values contained 2,686 proteins. FDR, False discovery rate, NM, normal mucosa; T, tumour; LM, liver metastasis


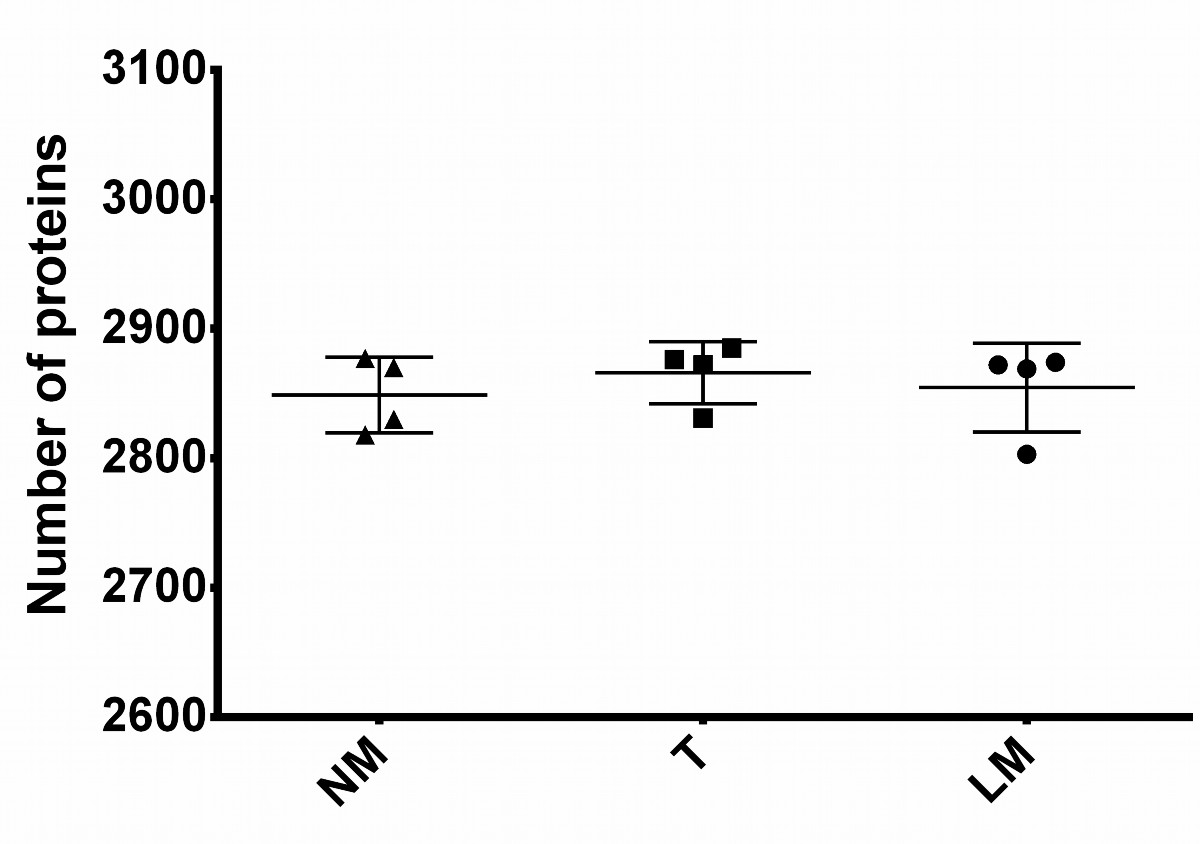


**Figure S4: Unsupervised principal component analysis of all three groups after two-dimensional gel electrophoresis.** PCA plot displays all four individual patients (P1, blue; P2, yellow; P3, purple; P4, grey). X- and y-axes show the first and second principal components, respectively.

**Figure S5: Panomics profiling of patient 1. Fold-change plots showing protein (a, b) and RNA (c, d) expression values for the NM1 vs T1 and NM1 vs LM1 (a, c) as well as for the T1 vs LM1 comparison (b, d).** FC-plots list the top 15 differentially expressed proteins/RNAs with either a higher or lower protein/gene expression, significantly expressed onco-proteins of the group comparisons, and significant targets of the group comparisons overlapping between gene and protein expression data (CD74, TNC, CEACAM5, CA1, CLCA1, MATN2, AHCYL2, FCGBP).

**
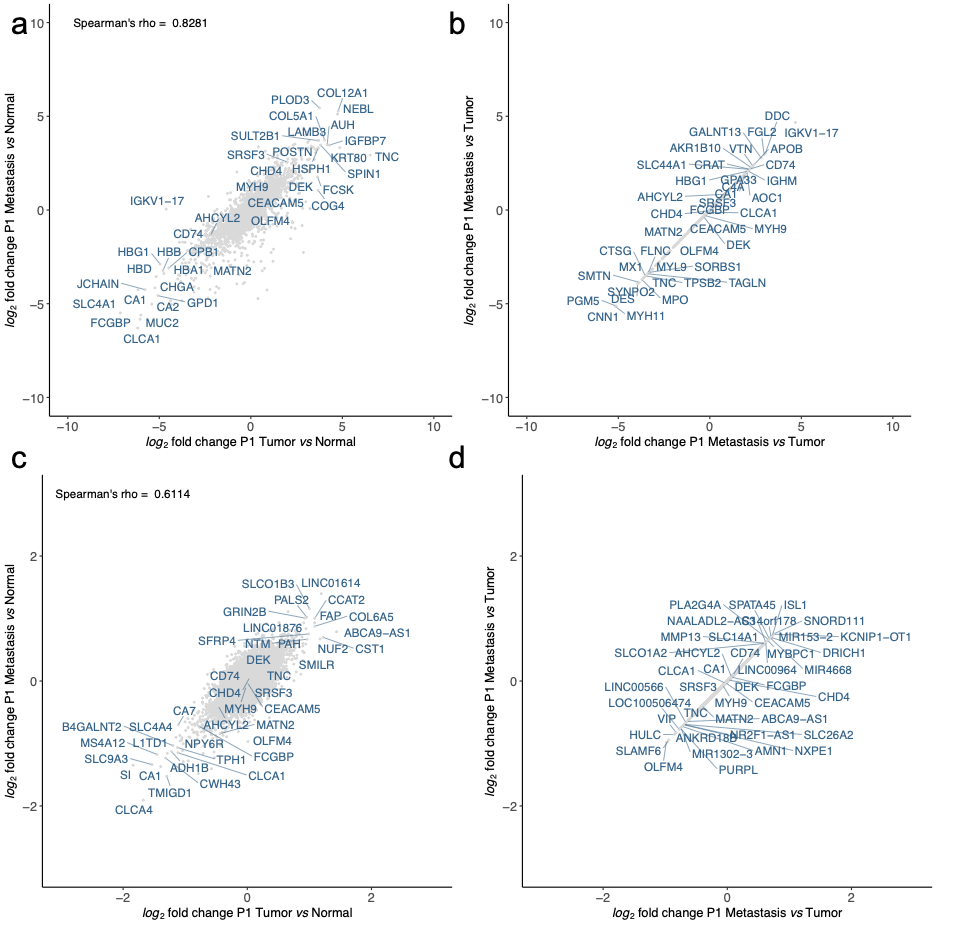
**

**Figure S6: Panomics profiling of patient 2. Fold-change plots showing protein (a, b) and RNA (c, d) expression values for the NM2 vs T2 and NM2 vs LM2 (a, c) as well as for the T2 vs LM2 comparison (b, d).** FC-plots list the top 15 differentially expressed proteins/RNAs with either a higher or lower protein/gene expression, significantly expressed onco-proteins of the group comparisons, and significant targets of the group comparisons overlapping between gene and protein expression data (SRSF3, CEACAM5, CA1, CLCA1, MATN2, AHCYL2, FCGBP).

**
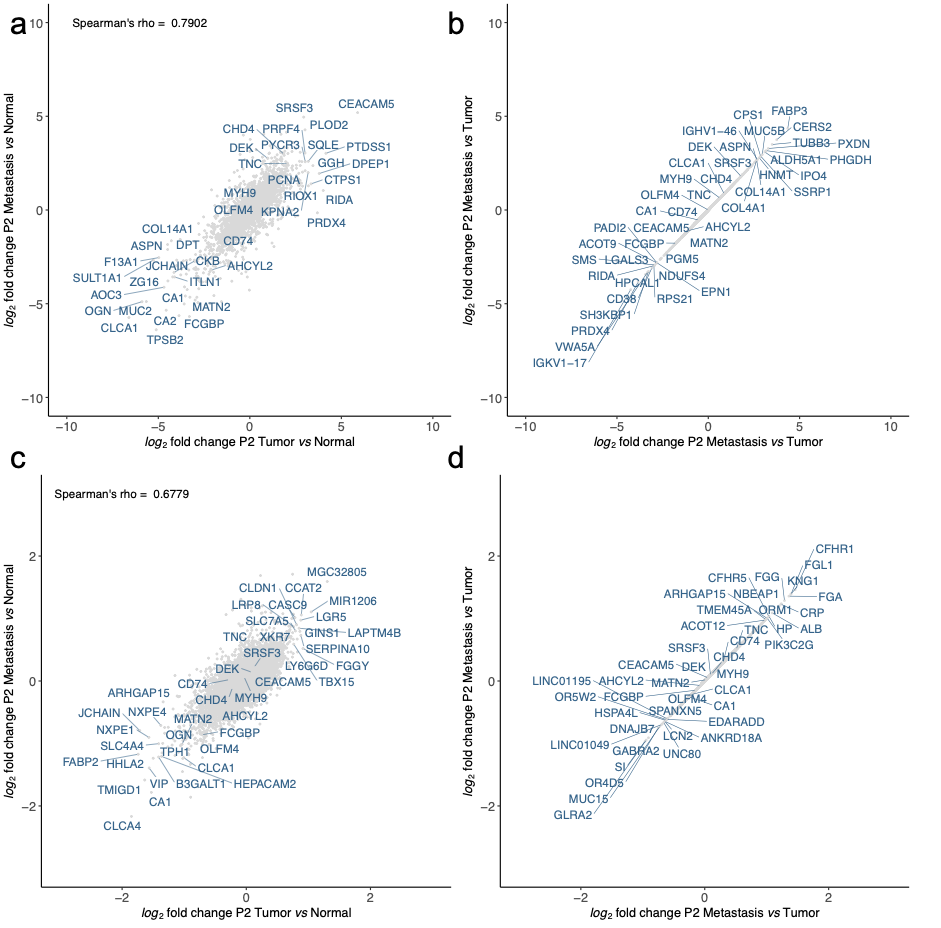
**

**Figure S7: Panomics profiling of patient 3. Fold-change plots showing protein (a, b) and RNA (c, d) expression values for the NM3 vs T3 and NM3 vs LM3 (a, c) as well as for the T3 vs LM3 comparison (b, d).** FC-plots list the top 15 differentially expressed proteins/RNAs with either a higher or lower protein/gene expression, significantly expressed onco-proteins of the group comparisons, and significant targets of the group comparisons overlapping between gene and protein expression data (CD74, TNC, CEACAM5, CA1, CLCA1, MATN2, AHCYL2, FCGBP).


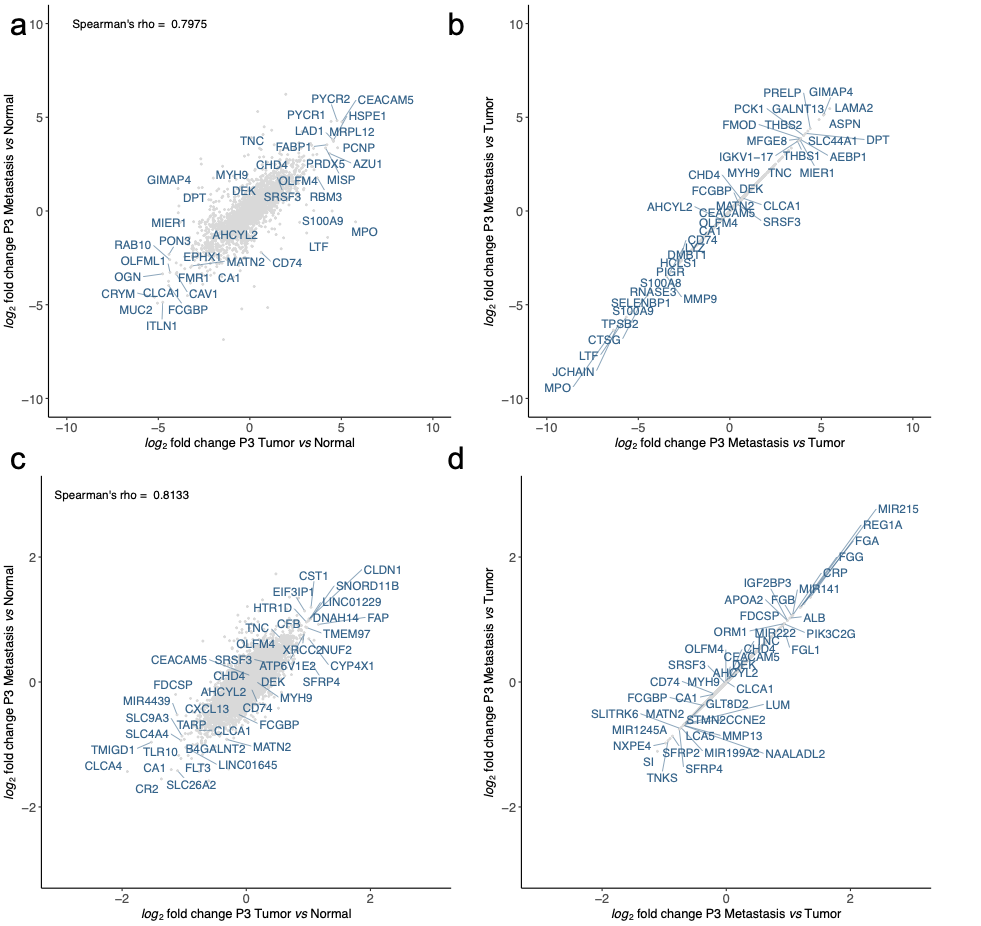


**Figure S8: Panomics profiling of patient 4. Fold-change plots showing protein (a, b) and RNA (c, d) expression values for the NM4 vs T4 and NM4 vs LM4 (a, c) as well as for the T4 vs LM4 comparison (b, d).** FC-plots list the top 15 differentially expressed proteins/RNAs with either a higher or lower protein/gene expression, significantly expressed onco-proteins of the group comparisons, and significant targets of the group comparisons overlapping between gene and protein expression data (DEK, CHD4, MYH9, CEACAM5, TNC, SRSF3, OLFM4, CA1, CLCA1, MATN2, AHCYL2, FCGBP).


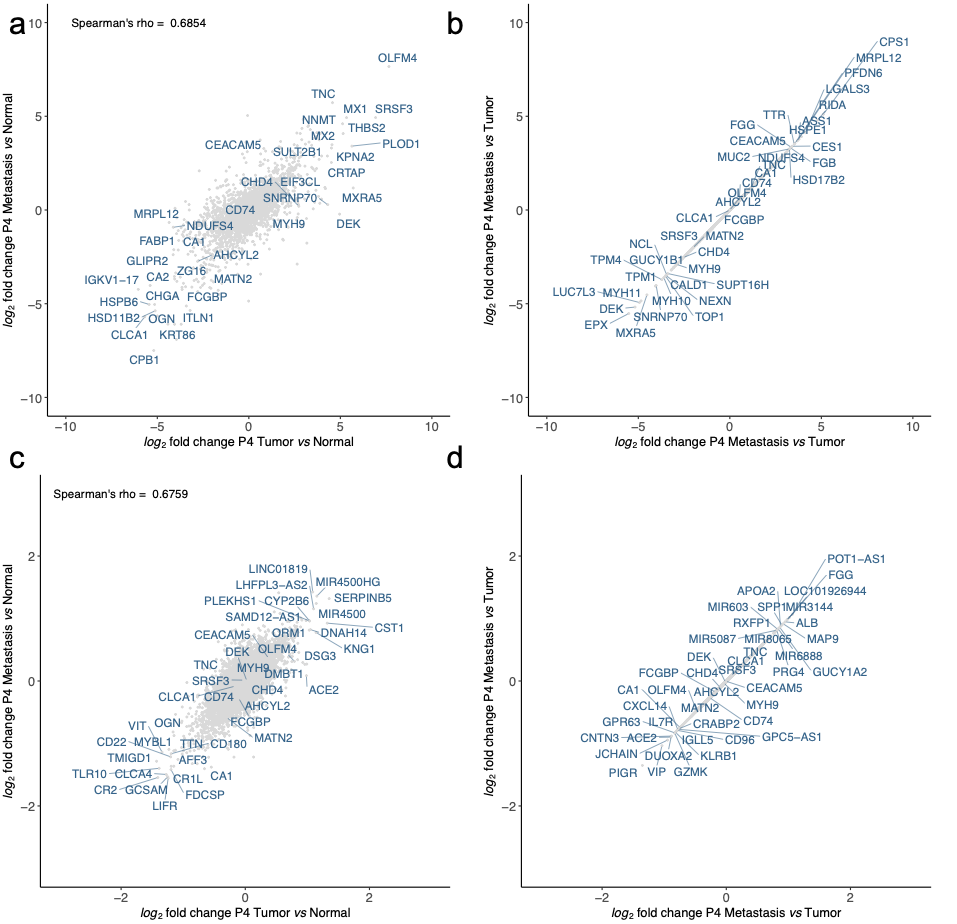


**Figure S9: Protein profiling of patient 4 showing six different tumour locations.** Fold-change plots showing protein expression values for the NM vs T_1-6_ and NM vs LM comparisons. FC-plots list the top 15 differentially expressed proteins with either a higher or lower gene expression, significantly expressed onco-proteins of the group comparisons, and significant targets of the group comparisons overlapping between gene and protein expression data.


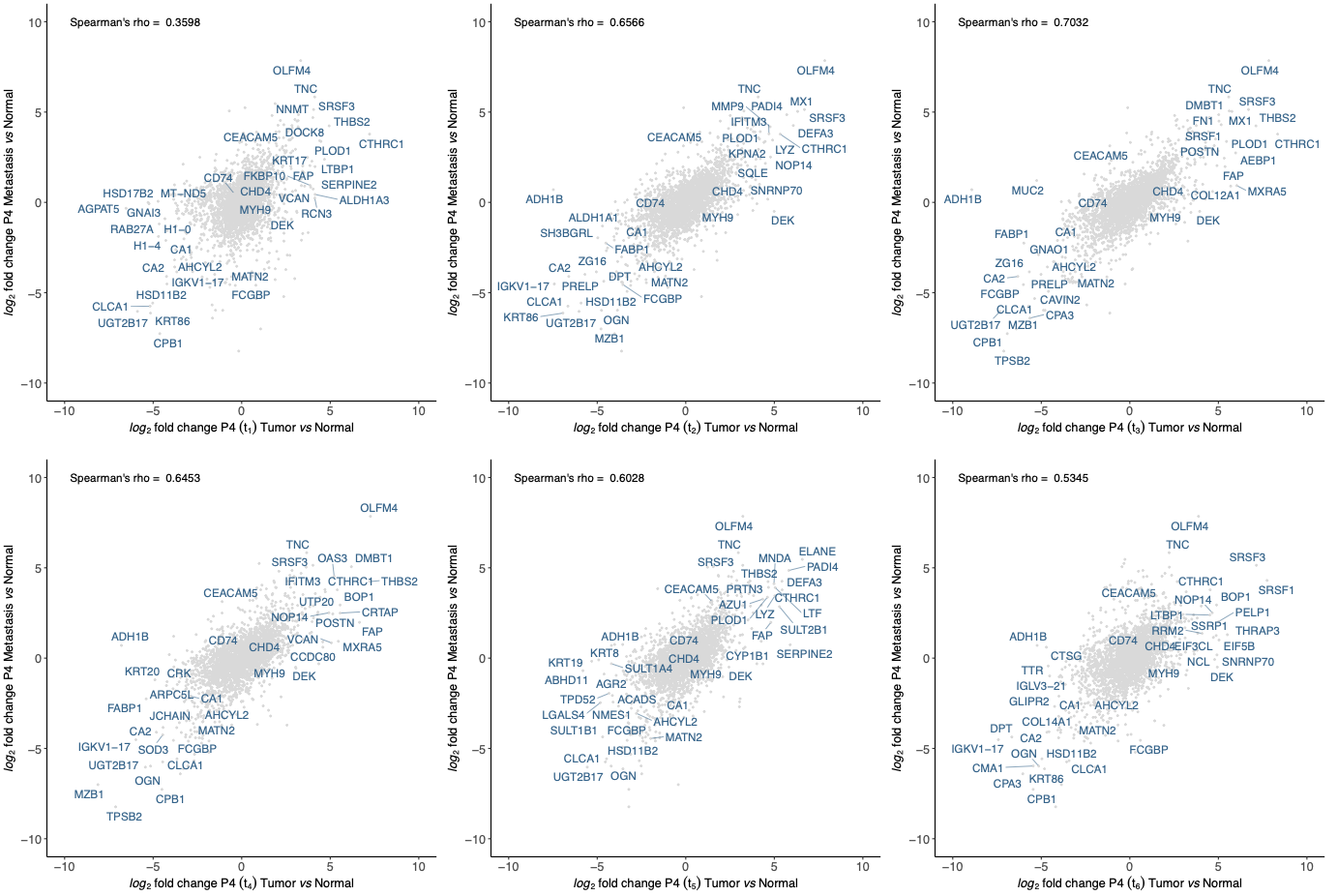


**Figure S10: Transcriptomic profiling of patient 4 showing six different tumour locations.** Fold-change plots showing gene expression values for the NM vs T_1-6_ and NM vs LM comparisons. FC-plots list the top 15 differentially expressed RNAs with either a higher or lower gene expression, significantly expressed onco-proteins of the group comparisons, and significant targets of the group comparisons overlapping between gene and protein expression data.


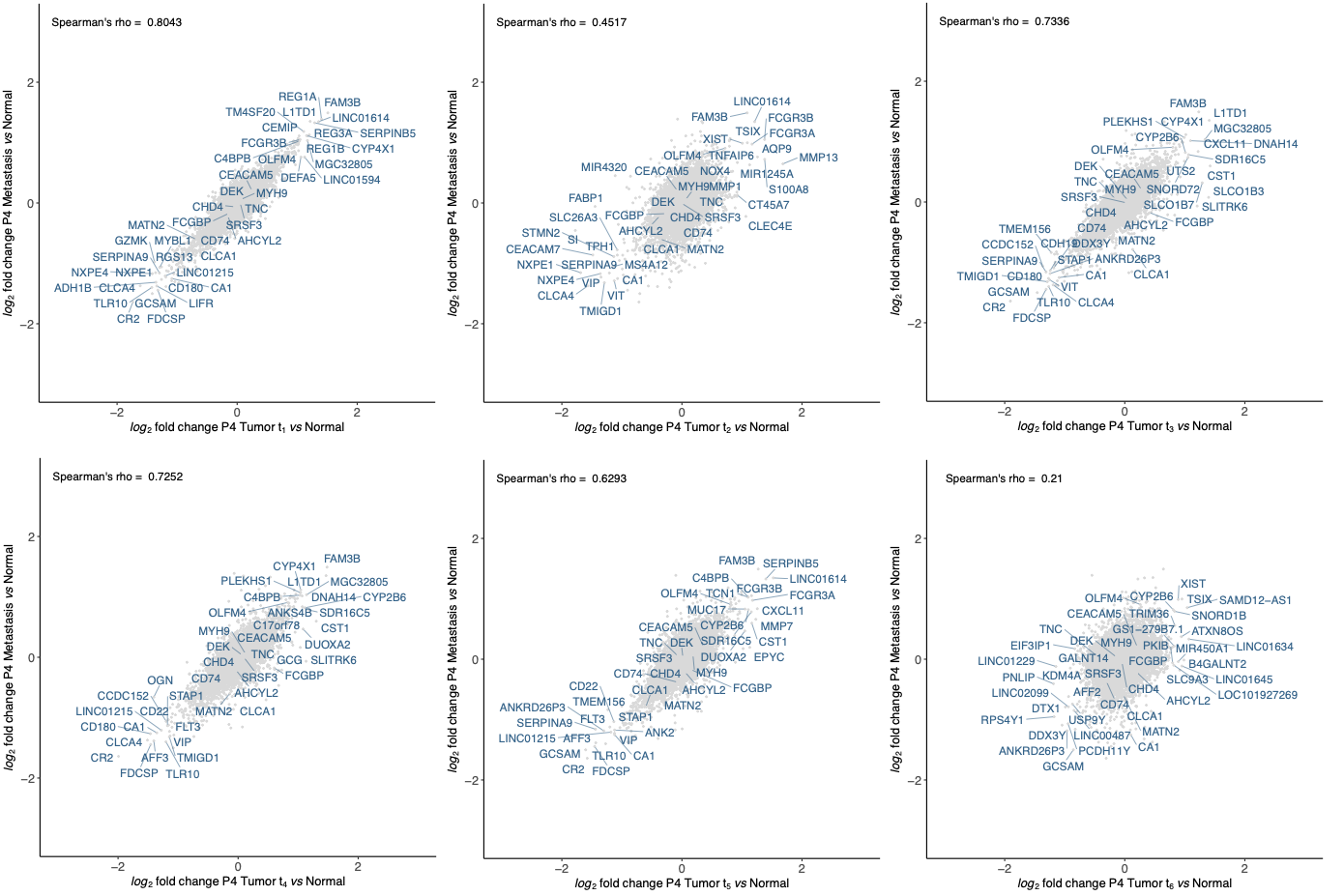


**Figure S11*:* Oncoplot depicting 17 detectable mutated genes of different tumour locations in P4 sorted and ordered by decreasing frequency.** T, tumour; M, liver metastasis
