# Supplementary material for: Panomics reveals patient individuality as the major driver of colorectal cancer progression
Source: J Transl Med. 2023 Jan 23;21:41. doi: 10.1186/s12967-022-03855-0 (PMC9869555; doi:10.1186/s12967-022-03855-0)
Supplement: Supplementary file 2 — Additional file 2: Table S1. Top 30 differentially expressed proteins in two-group comparisons of patient 1. Table S2. Top 30 differentially expressed RNAs in two-group comparisons of patient 1. Table S3. Top 30 differentially expressed proteins in two-group comparisons of patient 2. Table S4. Top 30 differentially expressed RNAs in two-group comparisons of patient 2. Table S5. Top 30 differentially expressed proteins in two-group comparisons of patient 3. Listed are the top 15 proteins with increased or decreased expression in the comparison of tumour vs. normal tissue, metastasis vs. normal tissue, and metastasis vs. tumour tissue measured by log2FC. Proteins marked in bold are products of onco- or tumour suppressor genes annotated in NCG 7.0. Table S6. Top 30 differentially expressed RNAs in two-group comparisons of patient 3. Listed are the top 15 RNAs with increased or decreased expression in the comparison of tumour vs. normal tissue, metastasis vs. normal tissue, and metastasis vs. tumour tissue measured by log2FC. RNAs marked in bold are onco- or tumour suppressor genes annotated in NCG 7.0. Table S7. Top 30 differentially expressed proteins in two-group comparisons of patient 4. Listed are the top 15 proteins with increased or decreased expression in the comparison of tumour vs. normal tissue, metastasis vs. normal tissue, and metastasis vs. tumour tissue measured by log2FC. Proteins marked in bold are products of onco- or tumour suppressor genes annotated in NCG 7.0. Table S8. Top 30 differentially expressed RNAs in two-group comparisons of patient 4. Listed are the top 15 RNAs with increased or decreased expression in the comparison of tumour vs. normal tissue, metastasis vs. normal tissue, and metastasis vs. tumour tissue measured by log2FC. RNAs marked in bold are onco- or tumour suppressor genes annotated in NCG 7.0. [file 12967_2022_3855_MOESM2_ESM.docx]

**Table S1: Top 30 differentially expressed proteins in two-group comparisons of patient 1.** Listed are the top 15 proteins with increased or decreased expression in the comparison of tumour vs. normal tissue, metastasis vs. normal tissue, and metastasis vs. tumour tissue measured by log_2_FC. Proteins marked in bold are products of onco- or tumour suppressor genes annotated in NCG 7.0.

| **T vs. NM** | **LM vs. NM** | **LM vs. T** |
| --- | --- | --- |
| **Top 15 proteins with increased expression** | | |
| **TNC** (6.52) | PLOD3 (5.45) | IGKV1-17 (4.67) |
| KRT80 (4.86) | COL12A1 (5.27) | DDC (3.04) |
| COL12A1 (4.77) | NEBL (5.12) | FGL2 (2.97) |
| NEBL (4.74) | AEBP1 (4.29) | GALNT13 (2.79) |
| IGFBP7 (4.28) | **SYK** (4.17) | APOB (2.44) |
| AUH (4.17) | NQO1 (4.02) | VTN (2.35) |
| COL5A1 (4.03) | S100A11 (3.98) | AKR1B10 (2.26) |
| SPIN1 (3.81) | THBS1 (3.97) | **CD74** (2.18) |
| PLOD3 (3.75) | COL5A1 (3.73) | CRAT (2.17) |
| LAMB3 (3.72) | LAMB3 (3.72) | SLC44A1 (2.17) |
| HSPH1 (3.69) | SULT2B1 (3.71) | IGHM (2.08) |
| COG4 (3.67) | TGFBI (3.70) | HBG1 (2.07) |
| FCSK (3.65) | PDP1 (3.61) | AOC1 (2.03) |
| POSTN (3.65) | MEP1A (3.52) | GPA33 (2.00) |
| SULT2B1 (3.65) | AUH (3.48) | C4A (1.91) |
| **Top 15 proteins with decreased expression** | | |
| SLC4A1 (-7.13) | CLCA1 (-6.30) | MYH11 (-5.15) |
| CLCA1 (-6.18) | FCGBP (-5.82) | CNN1 (-5.13) |
| CA1 (-6.17) | MUC2 (-5.60) | PGM5 (-5.12) |
| FCGBP (-6.05) | SLC4A1 (-5.49) | DES (-3.95) |
| MUC2 (-6.01) | FABP4 (-5.05) | SYNPO2 (-3.87) |
| JCHAIN (-5.78) | CA2 (-5.02) | SMTN (-3.86) |
| CA2 (-5.41) | PIGR (-4.84) | MPO (-3.71) |
| CHGA (-5.23) | CAV1 (-4.83) | MX1 (-3.63) |
| HBD (-5.17) | PGM5 (-4.73) | **TNC** (-3.59) |
| GPD1 (-5.08) | FHL1 (-4.70) | CTSG (-3.52) |
| HBG1 (-4.96) | GPD1 (-4.57) | TPSB2 (-3.51) |
| HBB (-4.80) | CAVIN2 (-4.41) | TAGLN (-3.40) |
| HBA1 (-4.75) | EPX (-4.38) | FLNC (-3.37) |
| IGKV1-17 (-4.63) | MYH11 (-4.27) | SORBS1 (-3.33) |
| CPB1 (-4.48) | CA1 (-4.26) | MYL9 (-3.30) |
| FC, fold change; NCG, Network of Cancer Genes, NM, normal mucosa; T, tumour; LM, liver metastasis | | |

**Table S2: Top 30 differentially expressed RNAs in two-group comparisons of patient 1.** Listed are the top 15 RNAs with increased or decreased expression in the comparison of tumour vs. normal tissue, metastasis vs. normal tissue, and metastasis vs. tumour tissue measured by log_2_FC. RNAs marked in bold are onco- or tumour suppressor genes annotated in NCG 7.0.

| **T vs. NM** | **LM vs. NM** | **LM vs. T** |
| --- | --- | --- |
| **Top 15 RNAs with increased expression** | | |
| ABCA9-AS1 (1.44) | LINC01614 (1.40) | SNORD111 (0.76) |
| CST1 (1.22) | SLCO1B3 (1.16) | KCNIP1-OT1 (0.75) |
| LINC01614 (1.19) | IL6 (1.11) | ISL1 (0.72) |
| NUF2 (1.17) | PALS2 (1.02) | MIR153-2 (0.72) |
| COL6A5 (1.09) | GRIN2B (1.01) | C14orf178 (0.70) |
| CCAT2 (1.09) | CCAT2 (1.00) | DRICH1 (0.70) |
| FAP (1.08) | MIR6888 (0.93) | MIR4668 (0.69) |
| SLCO1B3 (1.00) | FAP (0.93) | SPATA45 (0.68) |
| **SFRP4** (0.99) | MIR548AR (0.93) | PLA2G4A (0.66) |
| NTM (0.96) | SLC14A1 (0.92) | MYBPC1 (0.65) |
| PALS2 (0.96) | LOC101927967 (0.90) | NAALADL2-AS3 (0.62) |
| LINC01876 (0.95) | CLDN10-AS1 (0.88) | LINC00964 (0.62) |
| PAH (0.94) | COL6A5 (0.88) | SLC14A1 (0.61) |
| GRIN2B (0.92) | SERPINE1 (0.84) | MMP13 (0.60) |
| SMILR (0.92) | LINC01876 (0.84) | SLCO1A2 (0.60) |
| **Top 15 RNAs with decreased expression** | | |
| SI (-1.84) | CLCA4 (-1.91) | OLFM4 (-0.98) |
| CLCA4 (-1.68) | TMIGD1 (-1.52) | SLAMF6 (-0.94) |
| SLC9A3 (-1.53) | SLC26A2 (-1.48) | HULC (-0.85) |
| MS4A12 (-1.45) | VIP (-1.40) | VIP (-0.83) |
| CA1 (-1.39) | CA1 (-1.37) | LOC100506474 (-0.77) |
| CWH43 (-1.32) | SI (-1.35) | PURPL (-0.75) |
| TMIGD1 (-1.30) | SLC9A3 (-1.34) | MIR1302-3 (-0.75) |
| L1TD1 (-1.29) | HEPACAM2 (-1.29) | ANKRD18B (-0.74) |
| ADH1B (-1.22) | PIGR (-1.28) | AMN1 (-0.73) |
| B4GALNT2 (-1.20) | CWH43 (-1.23) | NXPE1 (-0.70) |
| SLC4A4 (-1.15) | NXPE1 (-1.23) | NR2F1-AS1 (-0.70) |
| TPH1 (-1.12) | MS4A12 (-1.18) | SLC26A2 (-0.69) |
| CLCA1 (-1.12) | REP15 (-1.16) | LINC00566 (-0.67) |
| CA7 (-1.11) | L1TD1 (-1.16) | ABCA9-AS1 (-0.65) |
| NPY6R (-1.09) | CLCA1 (-1.15) | **TNC** (-0.64) |
| FC, fold change; NCG, Network of Cancer Genes, NM, normal mucosa; T, tumour; LM, liver metastasis | | |

**Table S3: Top 30 differentially expressed proteins in two-group comparisons of patient 2.** Listed are the top 15 proteins with increased or decreased expression in the comparison of tumour vs. normal tissue, metastasis vs. normal tissue, and metastasis vs. tumour tissue measured by log_2_FC. Proteins marked in bold are products of onco- or tumour suppressor genes annotated in NCG 7.0.

| **T vs. NM** | **LM vs. NM** | **LM vs. T** |
| --- | --- | --- |
| **Top 15 proteins with increased expression** | | |
| CEACAM5 (5.89) | IPO4 (5.53) | FABP3 (4.35) |
| PTDSS1 (4.16) | CEACAM5 (5.19) | CERS2 (3.75) |
| RIDA (4.01) | **SRSF3** (4.96) | TUBB3 (3.50) |
| DPEP1 (3.79) | PLOD2 (4.28) | PXDN (3.28) |
| PRDX4 (3.69) | SUPT16H (4.15) | PHGDH (3.17) |
| GGH (3.45) | PXDN (4.05) | ALDH5A1 (3.12) |
| CTPS1 (3.34) | **CNBP** (4.04) | IPO4 (3.09) |
| RIOX1 (3.20) | FABP3 (3.99) | MUC5B (2.96) |
| SQLE (3.19) | CERS2 (3.77) | SSRP1 (2.87) |
| KPNA2 (3.19) | UTP20 (3.61) | CPS1 (2.87) |
| PYCR3 (3.03) | HDAC2 (3.43) | HNMT (2.79) |
| PCNA (3.02) | AGXT (3.35) | IGHV1-46 (2.69) |
| PLOD2 (3.02) | BYSL (3.31) | ASPN (2.65) |
| PRPF4 (3.01) | AEBP1 (3.31) | COL14A1 (2.65) |
| **SRSF3** (2.94) | DDX27 (3.27) | COL4A1 (2.64) |
| **Top 15 proteins with decreased expression** | | |
| CLCA1 (-6.61) | TPSB2 (-6.39) | IGKV1-17 (-4.25) |
| OGN (-5.89) | CLCA1 (-5.74) | PRDX4 (-3.84) |
| MUC2 (-5.65) | CHGA (-5.68) | HPCAL1 (-3.44) |
| SULT1A1 (-5.27) | FCGBP (-5.64) | VWA5A (-3.33) |
| TPSB2 (-5.11) | CA2 (-5.35) | SH3KBP1 (-3.27) |
| F13A1 (-4.97) | CPA3 (-5.32) | CD38 (-3.09) |
| AOC3 (-4.69) | PGM5 (-5.05) | RPS21 (-2.98) |
| CA2 (-4.57) | CRYAB (-5.01) | RIDA (-2.97) |
| ASPN (-4.49) | PADI2 (-4.99) | NDUFS4 (-2.94) |
| DPT (-4.49) | KRT86 (-4.94) | SMS (-2.93) |
| ZG16 (-4.47) | OGN (-4.89) | LGALS3 (-2.84) |
| COL14A1 (-4.29) | VWA5A (-4.89) | ACOT9 (-2.82) |
| JCHAIN (-4.21) | MUC2 (-4.89) | PADI2 (-2.82) |
| CKB (-4.14) | SULT1B1 (-4.80) | EPN1 (-2.81) |
| ITLN1 (-4.11) | CAVIN2 (-4.66) | PGM5 (-2.64) |
| FC, fold change; NCG, Network of Cancer Genes, NM, normal mucosa; T, tumour; LM, liver metastasis | | |

**Table S4: Top 30 differentially expressed RNAs in two-group comparisons of patient 2.** Listed are the top 15 RNAs with increased or decreased expression in the comparison of tumour vs. normal tissue, metastasis vs. normal tissue, and metastasis vs. tumour tissue measured by log_2_FC. RNAs marked in bold are onco- or tumour suppressor genes annotated in NCG 7.0.

| **T vs. NM** | **LM vs. NM** | **LM vs. T** |
| --- | --- | --- |
| **Top 15 RNAs with increased expression** | | |
| MGC32805 (1.30) | APOA2 (1.71) | CFHR1 (1.46) |
| MIR1206 (1.04) | CFHR1 (1.69) | FGL1 (1.40) |
| FGGY (0.91) | MGC32805 (1.59) | KNG1 (1.39) |
| CCAT2 (0.89) | ORM1 (1.47) | FGA (1.36) |
| SERPINA10 (0.88) | FGL1 (1.31) | FGG (1.29) |
| LGR5 (0.88) | CRP (1.22) | NBEAP1 (1.24) |
| GINS1 (0.86) | FGG (1.22) | CRP (1.23) |
| LAPTM4B (0.85) | FGB (1.20) | ORM1 (1.20) |
| LY6G6D (0.82) | ALB (1.17) | ALB (1.09) |
| XKR7 (0.82) | CFHR2 (1.17) | HP (1.04) |
| CLDN1 (0.81) | FGA (1.14) | CFHR5 (1.03) |
| SLC7A5 (0.79) | HP (1.12) | PIK3C2G (1.01) |
| CASC9 (0.78) | MIR1206 (1.11) | ARHGAP15 (1.01) |
| TBX15 (0.76) | ABCA9-AS1 (1.10) | TMEM45A (0.98) |
| LRP8 (0.76) | ORM2 (1.09) | ACOT12 (0.98) |
| **Top 15 RNAs with decreased expression** | | |
| CLCA4 (-1.85) | CLCA4 (-2.17) | SI (-0.96) |
| FABP2 (-1.74) | SI (-1.86) | GLRA2 (-0.94) |
| JCHAIN (-1.72) | CA1 (-1.78) | GABRA2 (-0.91) |
| TMIGD1 (-1.64) | TMIGD1 (-1.58) | OR4D5 (-0.75) |
| NXPE1 (-1.57) | SULT1B1 (-1.45) | MUC15 (-0.72) |
| VIP (-1.56) | VIP (-1.39) | LINC01049 (-0.70) |
| CA1 (-1.53) | SLITRK6 (-1.33) | DNAJB7 (-0.69) |
| HHLA2 (-1.50) | ABCG2 (-1.24) | UNC80 (-0.66) |
| B3GALT1 (-1.41) | HHLA2 (-1.24) | LCN2 (-0.65) |
| SLC4A4 (-1.41) | PKIB (-1.23) | ANKRD18A (-0.65) |
| NXPE4 (-1.38) | CLCA1 (-1.23) | HSPA4L (-0.63) |
| HEPACAM2 (-1.37) | B3GALT1 (-1.22) | OR5W2 (-0.62) |
| TPH1 (-1.36) | HEPACAM2 (-1.21) | EDARADD (-0.61) |
| OGN (-1.35) | MUC2 (-1.19) | LINC01195 (-0.61) |
| ARHGAP15 (-1.34) | MIR8065 (-1.18) | SPANXN5 (-0.60) |
| FC, fold change; NCG, Network of Cancer Genes, NM, normal mucosa; T, tumour; LM, liver metastasis | | |

**Table S5: Top 30 differentially expressed proteins in two-group comparisons of patient 3.** Listed are the top 15 proteins with increased or decreased expression in the comparison of tumour vs. normal tissue, metastasis vs. normal tissue, and metastasis vs. tumour tissue measured by log_2_FC. Proteins marked in bold are products of onco- or tumour suppressor genes annotated in NCG 7.0.

| **T vs. NM** | **LM vs. NM** | **LM vs. T** |
| --- | --- | --- |
| **Top 15 proteins with increased expression** | | |
| MPO (5.78) | THBS2 (6.23) | LAMA2 (5.46) |
| CEACAM5 (5.03) | GALNT13 (5.31) | ASPN (5.16) |
| PCNP (4.80) | PYCR2 (4.82) | GIMAP4 (5.12) |
| PYCR2 (4.76) | PYCR1 (4.78) | GALNT13 (4.88) |
| HSPE1 (4.57) | CEACAM5 (4.75) | PRELP (4.40) |
| MRPL12 (4.57) | THBS1 (4.49) | THBS2 (4.26) |
| LAD1 (4.51) | COL12A1 (4.31) | DPT (4.14) |
| S100A9 (4.50) | GPX2 (4.24) | SLC44A1 (4.04) |
| PYCR1 (4.44) | IGFBP7 (4.08) | PCK1 (4.03) |
| AZU1 (4.35) | AEBP1 (3.92) | FMOD (3.87) |
| LTF (4.25) | LAD1 (3.82) | MFGE8 (3.85) |
| FABP1 (4.21) | HSPE1 (3.77) | AEBP1 (3.83) |
| MISP (4.14) | MRPL12 (3.71) | MIER1 (3.82) |
| RBM3 (3.69) | BAIAP2L1 (3.64) | IGKV1-17 (3.75) |
| PRDX5 (3.66) | SMAP (3.60) | THBS1 (3.71) |
| **Top 15 proteins with decreased expression** | | |
| CRYM (-5.21) | TPSB2 (-6.86) | MPO (-6.36) |
| MUC2 (-5.05) | CTSG (-5.22) | JCHAIN (-6.13) |
| OGN (-4.76) | JCHAIN (-5.15) | LTF (-5.66) |
| ITLN1 (-4.76) | CPA3 (-5.07) | TPSB2 (-5.42) |
| CLCA1 (-4.44) | MUC2 (-4.92) | CTSG (-4.79) |
| FCGBP (-4.43) | ITLN1 (-4.87) | S100A9 (-4.50) |
| PON3 (-4.42) | CRYM (-4.60) | SELENBP1 (-4.28) |
| RAB10 (-4.38) | PGM5 (-4.52) | RNASE3 (-4.26) |
| OLFML1 (-4.36) | ADAM10 (-4.27) | S100A8 (-3.73) |
| CAV1 (-4.03) | RNASE3 (-4.03) | MMP9 (-3.47) |
| EPHX1 (-4.02) | FCGBP (-3.98) | PIGR (-3.33) |
| FMR1 (-3.99) | SLC4A1 (-3.91) | LYZ (-3.28) |
| GIMAP4 (-3.92) | F13A1 (-3.85) | HCLS1 (-3.28) |
| MIER1 (-3.91) | CLCA1 (-3.75) | **CD74** (-2.82) |
| DPT (-3.87) | RAB1A (-3.52) | DMBT1 (-2.76) |
| FC, fold change; NCG, Network of Cancer Genes, NM, normal mucosa; T, tumour; LM, liver metastasis | | |

**Table S6: Top 30 differentially expressed RNAs in two-group comparisons of patient 3.** Listed are the top 15 RNAs with increased or decreased expression in the comparison of tumour vs. normal tissue, metastasis vs. normal tissue, and metastasis vs. tumour tissue measured by log_2_FC. RNAs marked in bold are onco- or tumour suppressor genes annotated in NCG 7.0.

| **T vs. NM** | **LM vs. NM** | **LM vs. T** |
| --- | --- | --- |
| **Top 15 RNAs with increased expression** | | |
| FAP (1.16) | REG1A (1.73) | MIR215 (1.35) |
| NUF2 (1.09) | SERPINB5 (1.36) | REG1A (1.25) |
| **SFRP4** (1.05) | NMUR2 (1.34) | FGA (1.21) |
| CST1 (1.04) | FGB (1.31) | FGG (1.19) |
| SNORD11B (1.04) | FGG (1.21) | CRP (1.07) |
| CLDN1 (1.01) | CST1 (1.20) | MIR141 (1.07) |
| CYP4X1 (1.01) | LINC01610 (1.17) | FGB (1.05) |
| LINC01229 (1.01) | ORM1 (1.14) | ALB (1.02) |
| DNAH14 (1.00) | EIF3IP1 (1.14) | IGF2BP3 (0.99) |
| TMEM97 (0.98) | FGA (1.07) | APOA2 (0.98) |
| HTR1D (0.97) | VNN1 (1.07) | FDCSP (0.94) |
| CFB (0.96) | MIR5087 (1.02) | ORM1 (0.94) |
| EIF3IP1 (0.94) | APOA2 (1.02) | PIK3C2G (0.93) |
| XRCC2 (0.93) | CLDN1 (1.01) | FGL1 (0.91) |
| ATP6V1E2 (0.92) | MIR301A (1.01) | MIR222 (0.90) |
| **Top 15 RNAs with decreased expression** | | |
| CLCA4 (-1.92) | NXPE4 (-1.58) | SI (-1.11) |
| TMIGD1 (-1.53) | CR2 (-1.55) | NXPE4 (-0.98) |
| CR2 (-1.37) | STMN2 (-1.44) | TNKS (-0.94) |
| CA1 (-1.21) | CLCA4 (-1.43) | MIR1245A (-0.91) |
| FDCSP (-1.14) | SLC26A2 (-1.42) | SFRP2 (-0.87) |
| MIR4439 (-1.11) | CA1 (-1.40) | **SFRP4** (-0.76) |
| SLC26A2 (-1.11) | SI (-1.39) | SLITRK6 (-0.74) |
| TLR10 (-1.09) | TRHDE (-1.33) | MIR199A2 (-0.73) |
| **FLT3** (-1.05) | **FLT3** (-1.22) | LCA5 (-0.70) |
| SLC4A4 (-1.05) | MS4A12 (-1.22) | NAALADL2 (-0.69) |
| SLC9A3 (-1.04) | VIP (-1.18) | MMP13 (-0.69) |
| B4GALNT2 (-1.00) | TLR10 (-1.18) | CCNE2 (-0.68) |
| TARP (-0.98) | TMEM236 (-1.16) | LUM (-0.68) |
| LINC01645 (-0.98) | SNORD114-12 (-1.05) | STMN2 (-0.68) |
| CXCL13 (-0.97) | LINC01645 (-1.04) | GLT8D2 (-0.67) |
| FC, fold change; NCG, Network of Cancer Genes, NM, normal mucosa; T, tumour; LM, liver metastasis | | |

**Table S7: Top 30 differentially expressed proteins in two-group comparisons of patient 4.** Listed are the top 15 proteins with increased or decreased expression in the comparison of tumour vs. normal tissue, metastasis vs. normal tissue, and metastasis vs. tumour tissue measured by log_2_FC. Proteins marked in bold are products of onco- or tumour suppressor genes annotated in NCG 7.0.

| **T vs. NM** | **LM vs. NM** | **LM vs. T** |
| --- | --- | --- |
| **Top 15 proteins with increased expression** | | |
| OLFM4 (7.65) | OLFM4 (7.65) | CPS1 (5.11) |
| **SRSF3** (6.93) | **TNC** (5.73) | RIDA (4.80) |
| MXRA5 (5.70) | CPS1 (5.04) | LGALS3 (4.12) |
| PLOD1 (5.63) | **SRSF3** (4.93) | HSPE1 (3.91) |
| MX1 (5.34) | MX1 (4.93) | ASS1 (3.78) |
| THBS2 (5.15) | DMBT1 (4.83) | MRPL12 (3.69) |
| NNMT (5.12) | NNMT (4.60) | PFDN6 (3.55) |
| **DEK** (4.96) | ISG15 (4.51) | TTR (3.52) |
| **TNC** (4.57) | FN1 (4.46) | CES1 (3.40) |
| MX2 (4.54) | OAS3 (4.35) | FGG (3.35) |
| KPNA2 (4.51) | MUC1 (4.29) | CEACAM5 (3.34) |
| CRTAP (4.51) | MMP9 (4.13) | FGB (3.31) |
| SNRNP70 (4.31) | HK3 (4.11) | MUC2 (3.26) |
| SULT2B1 (4.29) | THBS1 (4.08) | HSD17B2 (3.26) |
| EIF3CL (4.18) | THBS2 (4.08) | NDUFS4 (3.21) |
| **Top 15 proteins with decreased expression** | | |
| IGKV1-17 (-6.04) | CPB1 (-7.50) | EPX (-5.52) |
| CLCA1 (-5.69) | TPSB2 (-6.91) | **DEK** (-5.18) |
| HSPB6 (-5.45) | ITLN1 (-6.10) | LUC7L3 (-4.94) |
| CA2 (-5.39) | CPA1 (-6.10) | MYH11 (-4.85) |
| CPB1 (-5.20) | KRT86 (-6.09) | MXRA5 (-4.53) |
| CHGA (-5.15) | CLCA1 (-5.70) | TPM1 (-4.06) |
| HSD11B2 (-5.12) | OGN (-5.65) | SNRNP70 (-4.03) |
| OGN (-4.99) | HSD11B2 (-5.38) | TPM4 (-3.72) |
| KRT86 (-4.42) | DCN (-5.36) | MYH10 (-3.61) |
| MRPL12 (-4.35) | CAVIN2 (-5.12) | TOP1 (-3.52) |
| GLIPR2 (-4.13) | CHGA (-5.04) | NEXN (-3.49) |
| NDUFS4 (-4.13) | HSPB6 (-5.04) | NCL (-3.46) |
| FABP1 (-4.10) | MYH11 (-4.86) | CALD1 (-3.41) |
| ZG16 (-4.07) | CKB (-4.59) | SUPT16H (-3.39) |
| ITLN1 (-4.06) | AOC3 (-4.50) | GUCY1B1 (-3.21) |
| FC, fold change; NCG, Network of Cancer Genes, NM, normal mucosa; T, tumour; LM, liver metastasis | | |

**Table S8: Top 30 differentially expressed RNAs in two-group comparisons of patient 4.** Listed are the top 15 RNAs with increased or decreased expression in the comparison of tumour vs. normal tissue, metastasis vs. normal tissue, and metastasis vs. tumour tissue measured by log2FC. RNAs marked in bold are onco- or tumour suppressor genes annotated in NCG 7.0.

| **T vs. NM** | **LM vs. NM** | **LM vs. T** |
| --- | --- | --- |
| **Top 15 RNAs with increased expression** | | |
| SERPINB5 (1.35) | LINC01819 (1.43) | POT1-AS1 (1.06) |
| CST1 (1.32) | APOA2 (1.41) | FGG (1.01) |
| MIR4500HG (1.15) | MIR4500HG (1.36) | LOC101926944 (0.98) |
| MIR4500 (1.14) | SERPINB5 (1.32) | MIR3144 (0.98) |
| KNG1 (1.14) | APOH (1.30) | ALB (0.95) |
| LHFPL3-AS2 (1.10) | MIR4500 (1.24) | MAP9 (0.92) |
| LINC01819 (1.09) | LHFPL3-AS2 (1.16) | SPP1 (0.89) |
| DNAH14 (1.05) | DEFA5 (1.11) | APOA2 (0.87) |
| CYP2B6 (1.03) | LGR5 (1.08) | GUCY1A2 (0.86) |
| PLEKHS1 (1.03) | GRIN2B (1.01) | MIR6888 (0.84) |
| ORM1 (1.03) | LINC02315 (1.01) | MIR603 (0.83) |
| DSG3 (0.99) | RNF128 (0.97) | RXFP1 (0.83) |
| ACE2 (0.98) | SAMD12-AS1 (0.97) | MIR5087 (0.81) |
| SAMD12-AS1 (0.98) | CYP2B6 (0.96) | PRG4 (0.81) |
| DMBT1 (0.96) | PLEKHS1 (0.96) | MIR8065 (0.81) |
| **Top 15 RNAs with decreased expression** | | |
| TMIGD1 (-1.43) | LIFR (-1.56) | PIGR (-1.35) |
| CR2 (-1.41) | CR2 (-1.55) | VIP (-1.04) |
| TLR10 (-1.39) | GCSAM (-1.53) | JCHAIN (-0.95) |
| VIT (-1.34) | CLCA4 (-1.50) | ACE2 (-0.90) |
| CLCA4 (-1.27) | FDCSP (-1.42) | DUOXA2 (-0.89) |
| GCSAM (-1.25) | CA1 (-1.40) | CNTN3 (-0.88) |
| LIFR (-1.24) | TLR10 (-1.40) | GZMK (-0.83) |
| MYBL1 (-1.23) | GZMK (-1.38) | GPR63 (-0.83) |
| CD22 (-1.20) | **AFF3** (-1.37) | KLRB1 (-0.82) |
| FDCSP (-1.20) | CR1L (-1.36) | **IL7R** (-0.80) |
| CD180 (-1.19) | LINC01215 (-1.32) | IGLL5 (-0.79) |
| **AFF3** (-1.19) | ANK2 (-1.32) | CD96 (-0.79) |
| OGN (-1.16) | TMIGD1 (-1.28) | CXCL14 (-0.78) |
| CR1L (-1.15) | CCDC152 (-1.26) | GPC5-AS1 (-0.77) |
| TTN (-1.15) | STAP1 (-1.24) | CRABP2 (-0.77) |
| FC, fold change; NCG, Network of Cancer Genes, NM, normal mucosa; T, tumour; LM, liver metastasis | | |
